# Spatiotemporal patterns and environmental drivers of human echinococcoses over a twenty-year period in Ningxia Hui Autonomous Region, China

**DOI:** 10.1186/s13071-018-2693-z

**Published:** 2018-02-22

**Authors:** Angela M. Cadavid Restrepo, Yu Rong Yang, Donald P. McManus, Darren J. Gray, Tamsin S. Barnes, Gail M. Williams, Ricardo J. Soares Magalhães, Nicholas A. S. Hamm, Archie C. A. Clements

**Affiliations:** 1 Research School of Population Health, The Australian National University, Canberra, ACT Australia; 2 Ningxia Medical University, Yinchuan, Ningxia Hui Autonomous Region People’s Republic of China; 3 Molecular Parasitology Laboratory, QIMR Berghofer Medical Research Institute, Brisbane, QLD Australia; 4 School of Veterinary Science, The University of Queensland, Gatton, QLD Australia; 5 Queensland Alliance for Agriculture and Food Innovation, The University of Queensland, Gatton, QLD Australia; 6 School of Public Health, The University of Queensland, Brisbane, QLD Australia; 7 Children’s Health and Environment Program, Child Health Research Centre, The University of Queensland, Brisbane, QLD Australia; 8 Faculty of Geo-Information Science and Earth Observation (ITC), University of Twente, Enschede, The Netherlands

**Keywords:** Echinococcosis, Cystic echinococcosis, Alveolar echinococcosis, Spatial analysis, Environmental change, Remote sensing

## Abstract

**Background:**

Human cystic (CE) and alveolar (AE) echinococcoses are zoonotic parasitic diseases that can be influenced by environmental variability and change through effects on the parasites, animal intermediate and definitive hosts, and human populations. We aimed to assess and quantify the spatiotemporal patterns of human echinococcoses in Ningxia Hui Autonomous Region (NHAR), China between January 1994 and December 2013, and examine associations between these infections and indicators of environmental variability and change, including large-scale landscape regeneration undertaken by the Chinese authorities.

**Methods:**

Data on the number of human echinococcosis cases were obtained from a hospital-based retrospective survey conducted in NHAR for the period 1 January 1994 through 31 December 2013. High-resolution imagery from Landsat 4/5-TM and 8-OLI was used to create single date land cover maps. Meteorological data were also collected for the period January 1980 to December 2013 to derive time series of bioclimatic variables. A Bayesian spatio-temporal conditional autoregressive model was used to quantify the relationship between annual cases of CE and AE and environmental variables.

**Results:**

Annual CE incidence demonstrated a negative temporal trend and was positively associated with winter mean temperature at a 10-year lag. There was also a significant, nonlinear effect of annual mean temperature at 13-year lag. The findings also revealed a negative association between AE incidence with temporal moving averages of bareland/artificial surface coverage and annual mean temperature calculated for the period 11–15 years before diagnosis and winter mean temperature for the period 0–4 years. Unlike CE risk, the selected environmental covariates accounted for some of the spatial variation in the risk of AE.

**Conclusions:**

The present study contributes towards efforts to understand the role of environmental factors in determining the spatial heterogeneity of human echinococcoses. The identification of areas with high incidence of CE and AE may assist in the development and refinement of interventions for these diseases, and enhanced environmental change risk assessment.

**Electronic supplementary material:**

The online version of this article (10.1186/s13071-018-2693-z) contains supplementary material, which is available to authorized users.

## Background

Cystic (CE) and alveolar (AE) echinococcoses, caused by *Echinococcus granulosus* and *E. multilocularis*, respectively, are the two forms of human echinococcosis of major public health importance worldwide [1]. Both diseases are distributed widely and potentially life threatening if left untreated [2–4]. Within China, *E. granulosus* and *E. multilocularis* are responsible for approximately 0.6–1.3 million human cases, with transmission occurring predominantly in central and western areas. Based on reports from the Chinese Ministry of Health, more than 98% of patients with human echinococcoses originate from Gansu, Qinghai and Sichuan Provinces and from Xinjiang Uygur, Ningxia Hui and Inner Mongolia Autonomous Regions [5]. Although these regions constitute highly endemic areas for these diseases in East Asia, significant differences in parasite prevalences have been demonstrated at regional and local levels [6–8]. On the Qinghai-Tibet Plateau, where there is high transmission of *Echinococcus* spp., the prevalence of both CE and AE ranges between 0.4–9.5%, being higher in communities where pastoralism and poor socio-economic conditions are predominant [9, 10]. The patchy AE endemicity distribution has been associated with landscape characteristics and climatic factors that determine habitat suitability for the definitive and intermediate hosts [11–17]. Hence, understanding how environmental and social factors interact to determine parasite transmission is essential for the design and implementation of effective strategies against echinococcosis, and to target resources to the communities most in need.

*Echinococcus* spp. are maintained primarily through complex domestic and sylvatic life-cycles that involve a wide range of intermediate and definitive hosts and a free-living egg stage. Humans are accidental hosts, that acquire the infection through direct contact with definitive hosts or through a contaminated environment [2]. In the sylvatic and semi-domestic (*E. multilocularis*) and domestic (*E. granulosus*) life-cycles of the parasites, distinct socio-demographic and environmental factors modulate the parasite-host-human interplay at different spatial scales [18, 19]. Therefore, different processes of environmental change have the potential to modify the transmission pathways of these parasites [18].

Various land reform policies and incentive programmes have been developed in China to recover degraded lands and promote sustainable development in rural areas [20]. The Grain for Green Project (GGP), also called the Sloping Land Conversion Programme, implemented since 1999, is one of the largest payment for ecosystems services schemes in China [21]. The main focus of the programme is to rehabilitate the ecological environment by promoting three different types of land conversion on steep slopes: cropland to grasslands, cropland to forest and wasteland to forest [21]. The GGP also advocates for small ruminant enclosure and grazing prohibition. In highly endemic areas for echinococcoses, the anthropogenic-driven land cover modifications that resulted apparently from the implementation of the GGP and other reforestation programmes might have favoured the transmission of *E. multilocularis*. Evidence on the impact of deforestation [13, 22], afforestation [11] and fencing/agricultural practices [23–25] on the population density and distribution of small mammals is increasing.

Recognizing the public health and economic significance of human echinococcoses, and the potential risk of parasite range expansion, the National Health and Family Planning Commission (NHFPC) launched a national action plan for echinococcosis control in 2005 [26]. This initiative aims to decrease the seropositivity rate in children aged < 12 years and to reduce infestation rates in dogs. To achieve these goals, five interventions were designed to reduce the impacts of these infections in 217 endemic counties: community-based epidemiological surveys involving serological, abdominal ultrasound and chest X-ray screening for early detection of cases; treatment and surveillance of patients diagnosed with the disease; education campaigns to enhance awareness among local people and health officials; regular anti-helmintic treatment for deworming of dogs; and improved control of slaughter practices [27]. In general, the coordination of these efforts has proven difficult, especially in rural areas [26, 27]. In order to improve the establishment and monitoring of realistic targets for control, it is necessary to estimate the real impact of these infections and the permissive factors for transmission at local and regional scales [28].

Using geographical information systems (GIS), Earth observation data and a Bayesian statistical framework, the present study describes the spatio-temporal patterns of CE and AE in NHAR between January 1994 and December 2013. The aims were to identify highly endemic areas for these infections in the autonomous region, and to determine the environmental covariates that are shaping their local geographical distributions, in particular those that may be indicators of the potential impact of the GGP on the NHAR land cover profile. The findings may help the targeting of resources to communities most in need of echinococcosis control, and by contributing to environmental risk assessments of major landscape regeneration programmes such as the GGP.

## Methods

### Study area

NHAR is a province-level autonomous region located in Northwest China between latitudes 35°26′N and 39°30′N, and between longitudes 104°50′E and 107°40′E. The provincial territory covers an area of 66,400 km^2^ and is bordered by the Inner Mongolia Autonomous Region to the North, Gansu Province to the South and West and Shaanxi Province to the East. Administratively, NHAR is divided into 5 prefectures that are subsequently subdivided into counties/districts/county-level cities, townships and villages. The population reached about 6.6 million people in 2014, of whom the majority were living in urban areas (53.6%) compared to rural areas (46.4%) [29]. From those living in rural areas, 54.7% belonged to the Hui minority ethnic group and 45.3% were Han Chinese [29]. Internal migration, movement of people from one area (a province, prefecture, county or township) to another within one country [30], is particularly high in NHAR with 54.6% of households reporting at least one migrant in 2001 [31]. Also, a report from the Beijing Normal University and Hitotsubashi University in 2009 indicated that internal migration in NHAR increased from 17.2% in 2002 to 28.3% in 2008, among the working-age population (aged between 16 and 65 years) who participated in the GGP (participation period between 3 and 6 years), and decreased from 24% in 2002 to 17.6% in 2008 among people who did not participate in the programme [32]. The report also demonstrated that migration decision depends on various demographic and socioeconomic factors. In NHAR, migrants are young men with an education level of about 6–9 years, which coincides with the population with high tendency towards migration in China [33]. Variations in migration propensity between Han and Hui nationality groups or between married and non-married people were not found [32].

**Fig. 1:**
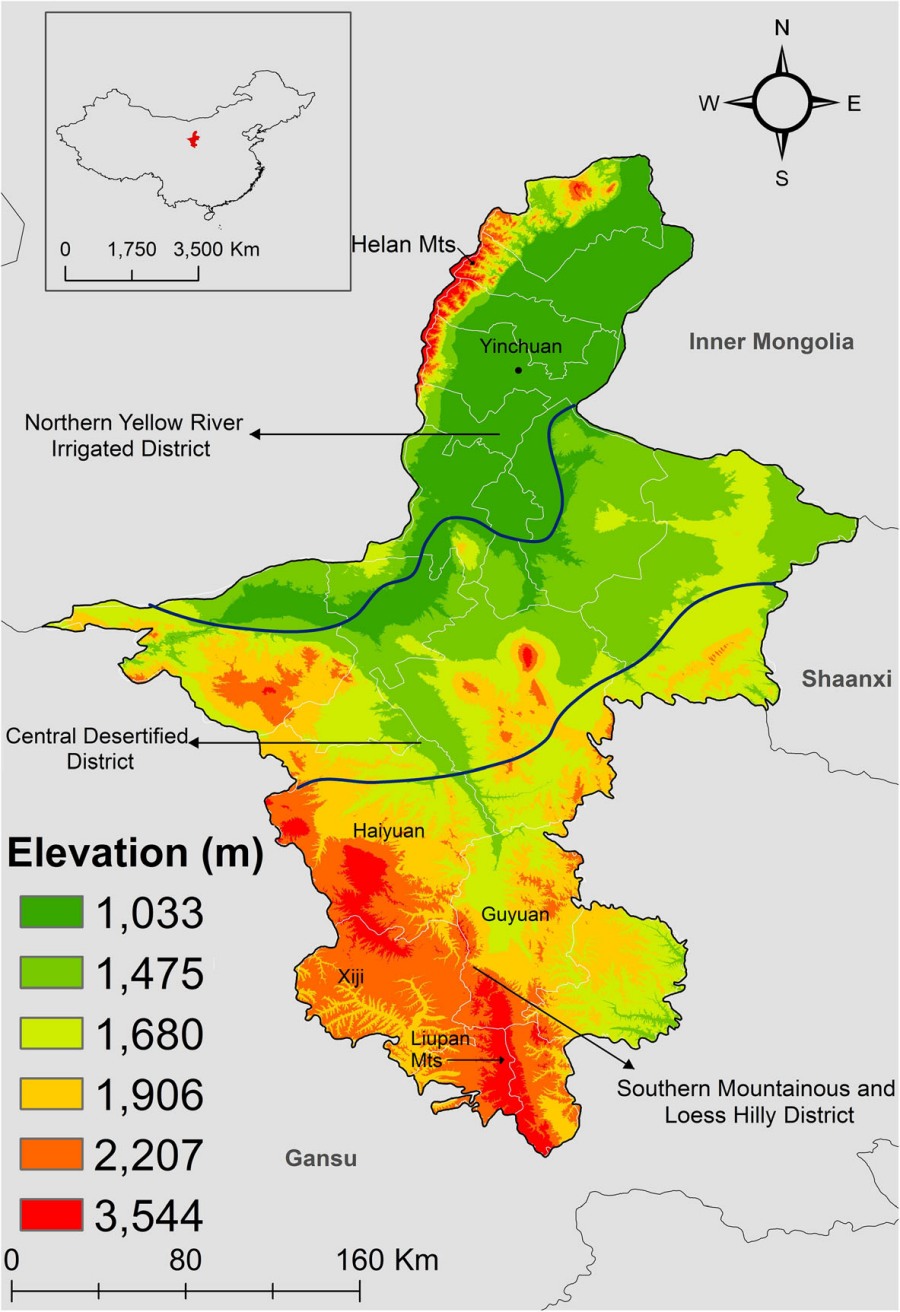
Map and elevation of NHAR and location of the province within China (insert). The blue lines divide the three major natural regions

NHAR lies in a temperate continental monsoon climate zone that is characterized by large seasonal variation in temperature, rainfall and humidity. About 80% of the annual rainfall occurs during the summer and autumn months and generally increases from North to South. Elevation increases from North to South with the highest peak at 3556 m (Fig. 1) [16].

### Data on human CE and AE

Data on the number of human CE and AE cases were derived from a hospital-based retrospective survey. Hospital medical records for the period between January 1 1992 and December 31 2013 were reviewed in 25 public hospitals in NHAR: 1 hospital from each county (*n* = 21), three hospitals from the capital city, Yinchuan, and 1 hospital from Guyuan Prefecture. Data collection was conducted during two different time periods, 2002–2003 and 2012–2013 and both involved the same number of hospitals. These hospitals were selected because they provide clinical and surgical care for most echinococcosis patients from rural and urban areas in the province. When patients with a presumptive echinococcosis diagnosis are admitted to local rural medical centres, they are usually referred to the county hospital for confirmation, treatment and follow-up examination. All patients whose diagnoses of CE and AE infection were established during the study period were included in the analysis. Inclusion criteria required that the diagnosis of a CE or AE case was confirmed based on imaging, serological, surgical and/or histopathologic findings. A standard form was used to extract individual information on relevant clinical, pathological and demographic data for all confirmed cases. Data were geo-referenced to the township in which each patient resided: this was assumed to be the geographical area where the infection occurred. The day of diagnosis was considered to be the date of primary surgical and confirmatory procedures. If a confirmed case was readmitted to hospital with the same diagnosis, only the initial admission was included in the analysis. The design and methods of the hospital survey for the period 1992–2002 have been described in detail elsewhere [34, 35]. The review of medical records for the period 2003–2013 followed the same protocol.

Because the data collected between 1992 and 1993 had considerable gaps, the CE and AE cases derived from these years were excluded from the analysis. For the purpose of our analyses the time period for the study was set from January 1 1994 to December 31 2013. To conduct the analysis, CE and AE cases were aggregated by township and year.

### Population data

Data on population for the year 2010 were downloaded from the WorldPop Project website [36]. A grid (i.e. raster surface) was available for the area of China at the resolution of 100 m. Population counts were extracted for each township using the ArcGIS software [37] and an administrative map of NHAR. In addition, data on population at the prefecture level were also obtained for the years 1990 and 2000 from the national censuses [38]. These data were used to calculate an average annual population growth rate for each prefecture between the years as follows: *r* = (*P*_*2*_*-P*_*1*_/*P*_*1*_)/*t*; where *r* is the average rate of growth, *P*_*1*_ and *P*_*2*_ are the population totals for the first and second reference years, respectively, and *t* is the number of years between the two census counts. Applying a Taylor series approximation to remove non-linear terms [39], the growth rate estimates were then used to calculate population counts for each township and year based on the 2010 population values derived from the WorldPop grid, (*P*_*2*_ *= P*_*1*_*e*^*(rt)*^) [40]. However, it should be noted that the approximation becomes increasingly erroneous as *t* increases (Additional file 1) [39].

### Climate and physical environment data

The independent variables included in the analysis were derived from the following datasets: land cover maps, elevation, monthly mean temperature and precipitation. Because data on human echinococcoses were collected for the period 1994–2013, the environmental datasets were derived from 1980 to 2013 to investigate environmental conditions that could account for exposures during the long incubation period that characterises echinococcosis infections (5–15 years) [41].

Single date land cover maps were created for the years 1991, 1996, 2000, 2005, 2010 and 2015. The scientific background and processing steps have already been published [9] so are only outlined in brief here. These maps were produced using images retrieved from the Landsat Surface Reflectance Climate Data Record available in Earth Explorer [42]. Four scenes processed from Landsat 4–5 Thematic Mapper and Landsat 8 Operational Land Imager and Thermal Infrared Sensor were collected for each year. Most scenes were retrieved from the summer and autumn season that correspond to the period June to November [43, 44]. When there were no scenes available for the selected months, the closest-in-time and most cloud-free scenes available were downloaded for the analyses. Minnaert topographic correction, cloud and cloud shadow removal were performed using the *Landsat* package in the R language and environment for statistical computing [45, 46]. Images were mosaicked and classified by applying the maximum likelihood algorithm in ENVI version 5.3 [47]. Reference datasets for land cover classification (training) were produced by random sampling of a combination of relatively fine-scale global maps, the GlobeLand30 and the global forest/non-forest maps (FNF) [48, 49] using the ArcGIS software version 10.3.1 [37]. Six land cover classes were identified: water bodies, artificial surfaces, bare or sparsely vegetated areas, herbaceous vegetation, cultivated land, shrubland and forest. Due to substantial similarities between the spectral values of artificial surfaces and bare or sparsely vegetated areas, these two classes were merged and represented as a single land cover category called bareland/artificial surfaces (Table 1).

**Table 1:** Land cover classification scheme and definitions

Land cover type	Description	Content
Water bodies	All areas of water	Streams and canals, lakes, reservoirs, bays and estuaries
Artificial surfaces	Land modified by human activities	Residential areas, industrial and commercial complexes, transport infrastructure, communications and utilities, mixed urban or built-up land and other built-up land
Bare or sparsely vegetated areas	Areas with little or no ‘green’ vegetation present	Dry salt flats, sandy areas, bared exposed rock and mixed barren land
Herbaceous vegetation	Areas characterized by natural or semi-natural vegetation	Grasses and forbs
Cultivated land	Areas where the natural vegetation has been removed/modified and replaced by other types of vegetative cover that have been planted for specific purposes such as food, feed and gardening	Cropland and pasture, orchards, groves, vineyards, nurseries and ornamental horticultural, other cultivated land
Shrubland	Natural or semi-natural woody vegetation with aerial stems less than 6 m tall	Evergreen and deciduous species of true shrubs and trees or shrubs that are small or stunted
Forest	Areas characterized by tree cover or semi-natural woody vegetation greater than 6 m tall	Deciduous forest, evergreen forest and mixed forest

Sets of space- and time-referenced photographs from the website Panoramio [50] were downloaded for each year to produce datasets for accuracy assessments of the land cover classes. In order to reduce the level of uncertainty due to the use of this type of data [51], all selected photographs were labelled manually based on visual interpretation, and cross-checked against historical imagery from Google Earth Pro (GEP) version 7.1.5.1557 [52]. The overall classification accuracies of all maps were higher or equal to 80% and the total Kappa coefficients were greater than 0.7. These results represent a substantial agreement between the reference datasets and the classified maps. The six land cover maps and more specific and detailed information about the process of land cover classification and validation is available elsewhere [53].

Monthly averages of temperature and precipitation data for the period January 1 1980 to December 31 2013 were provided by the Chinese Academy of Sciences. Data were first collected from 16 local weather stations and interpolated using the Inverse Distance Weighting (IDW) method. ESRI grids including the monthly data were obtained at the resolution of 1 km (approximately 30 arc-seconds) grid (Additional files 2, 3 and 4).

**Fig. 2:**
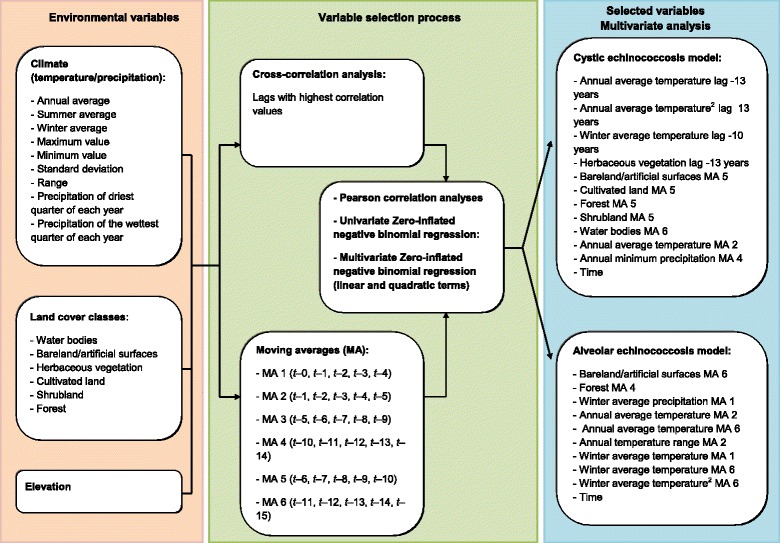
Environmental variables and variable selection process for the spatiotemporal analysis of human echinococcosis in NHAR for the period 1 January 1994 to 31 December 2013

Elevation data from the Advanced Spaceborne Thermal Emission and Reflection Radiometer (ASTER) Global Digital Elevation Model (GDEM) version 2 were downloaded from the USGS Earth Explorer website [54]. The ASTER GDEM is available globally in GeoTIFF format at the resolution of 1 arcsecond (approximately 30 m) (Fig. 2).

### Data analysis

A township-level shapefile (boundary map) of NHAR was produced using MapInfo Pro software version 15.0 [55] and a scanned and geo-referenced administrative map of NHAR provided by the Bureau of Geology and Mineral Resource. The administrative boundary map included 227 township-level areas. The small area of the townships (median 154 km^2^, interquartile range 73.5–297.5 km^2^) permitted an analysis of human echinococcoses at a high level of spatial disaggregation.

The spatial datasets including human echinococcosis cases, demographic and environmental data were imported into the ArcGIS software [37] and projected to the Universal Transverse Mercator (UTM) coordinate system zone 48 N. The datasets were linked according to location to the administrative boundary map of NHAR to summarise and extract the data by township area and define parameters for subsequent statistical analyses.

The spatial mean values of elevation and the spatial extent (as percentage of area) of each land cover class for the years 1991, 1996, 2000, 2005, 2010 and 2015 were calculated for all the townships. Because there were only six land cover maps, data extracted by class were then used to impute change rates at the township level for the periods 1991–1996, 1996–2000, 2000–2005, 2005–2010, and 2010–2015. In this way, it was possible to estimate the spatial extent of all land cover classes in each township for all years between 1980 and 2015.

Annual series of bioclimatic variables were calculated at the township level from the climate datasets. Monthly temperature and precipitation records were summed in the GIS to provide annual, summer (June-August) and winter (December-February) averages. Other variables that were calculated include maximum, minimum, standard deviation, range values and precipitation of the driest and wettest quarters of each year.

Crude standardised morbidity ratios (SMRs) for each administrative area were calculated for the periods 1994–1998, 1999–2003, 2004–2008 and 2009–2013. SMRs were computed by dividing the observed number of cases by the expected number of cases in the study population (overall incidence rate of human echinococcoses for the whole province from 1994 to 2013 multiplied by the population of each township).

To account for the long incubation period of CE and AE, temporal lags in the effects of land cover and bioclimatic variables were incorporated in the analysis by calculating cross-correlation coefficients between the CE and AE counts in a given year and the value of each environmental predictor at time *t* (*t*–0, *t*–1, *t*–2… *t*–34 years). From each bivariate time-lagged correlation, only the lag with the highest correlation value was selected for the analysis. A moving average (MA) technique was also applied to generate temporally smoothed estimates of the land cover and climate data. In this way, it was possible to capture the interplay between the parasite, hosts and the environment over an extended period of time rather than at a single point in time. In order to examine different short-, intermediate-and long-term exposure windows, the MAs were calculated by aggregating the environmental data in 5-year lagged periods as follows:

- MA 1 (*t*–0, *t*–1, *t*–2, *t*–3, *t*–4)
- MA 2 (*t*–1, *t*–2, *t*–3, *t*–4, *t*–5)
- MA 3 (*t*–5, *t*–6, *t*–7, *t*–8, *t*–9)
- MA 4 (*t*–10, *t*–11, *t*–12, *t*–13, *t*–14)
- MA 5 (*t*–6, *t*–7, *t*–8, *t*–9, *t*–10)
- MA 6 (*t*–11, *t*–12, *t*–13, *t*–14, *t*–15)

Univariate Zero-inflated negative binomial regression models were developed using the R software version 3.2.2. [45]. In this way, it was possible to assess separately the association of the response variables, CE and AE counts, with the environmental factors with the highest lagged correlation and all MAs. Zero-inflated negative binomial regression models were preferred over Poisson, negative binomial and zero-inflated Poisson models based on the results of the Vuong test [56]. Pearson correlation analyses were applied to assess collinearity among all environmental predictors. If the correlation coefficient between a pair of variables was > 0.9, the variable with the highest value of the Akaike information criterion (AIC) in the univariate regression model was excluded from the multivariate analysis. Nonlinear associations between the environmental covariates and CE/AE counts were also examined using quadratic terms (Fig. 2).

A Bayesian framework was used to construct three different Poisson regression models of the observed incidence data of CE and AE using the OpenBUGS software 3.2.3 rev 1012 [57]. The first model (Model I) was based on the assumption that spatial autocorrelation was not present in the relative risk of these infections. This model was developed incorporating time in years, the selected explanatory variables and an unstructured random effect for township; the second model (Model II) included the explanatory variables and a spatially structured random effect; the third model (Model III) was constructed without explanatory variables and incorporating only a spatially structured random effect (enabling an assessment of the degree to which the explanatory variables characterised spatial clustering of infections).

The mathematical notation for Model II is provided below, and contains all of the components of Model I and Model III. Model II, assumed that the observed counts of the infection (CE or AE), *Y*, for the ith township (*i* = 1.. .227) in the jth year (1994–2013) followed a Poisson distribution with mean (μ_ij_), that is,

$$ {Y}_i\sim Poisson\left({\mu}_{ij}\right) $$

$$ \log \left({\mu}_{ij}\right)=\log \left({Exp}_{ij}\right)+{\theta}_{ij} $$

$$ {\theta}_{ij}=\alpha +{\mathrm{Year}}_j\mathsf{x}\ \gamma +\sum \limits_{z=1}^z{\beta}_z\ \mathsf{x}\ {\uplambda}_{zij}+{s}_i $$

where *Exp*_*ij*_ is the expected number of cases in township *i* in year *j* (acting as an offset to control for population size) and *θ*_*ij*_ is the mean log relative risk (RR); *α* is the intercept, *γ* is the coefficient for temporal trend, *β* is a vector of z coefficients, *λ* is a matrix of z environmental covariates, and *s*_*i*_ is the spatially structured random effect with mean zero and variance *σ*_s_^2^. Standardization of environmental variables was used to allow comparability of the effects and provide a more meaningful interpretation on the results. Standardization, involved subtracting the mean from each environmental variable and the difference was divided by the standard deviation, which resulted in a standard deviation of one.

The spatially structured random effect (Models II and III) was modelled using a conditional autoregressive (CAR) prior structure [58]. This approach uses an adjacency weights matrix to determine spatial relationships between townships. If two townships share a border, it was assumed the weight = 1 and if they do not, the weight = 0. The adjacency matrix was constructed using the ‘Adjacency Tool’ of the OpenBUGS software 3.2.3 rev 1012 [57]. A flat prior distribution was specified for the intercept, whereas a normal prior distribution was used for the coefficients (with a mean = 0 and a precision = 0.001). The priors for the precision (1/*σ*_*t*_^*2*^) of spatially structured random effects were specified using non-informative gamma distributions (0.5, 0.0005) (Additional files 5 and 6).

The first 1000 iterations were run as a burn-in period and discarded. Subsequent sets of 20,000 iterations were run and examined for convergence. Convergence was determined by visual inspection of posterior density and history plots and by examining autocorrelation plots of model parameters. Convergence occurred at approximately 100,000 iterations for each model. The last 20,000 values from the posterior distributions of each model parameters were stored and summarised for the analysis. The deviance information criterion (DIC) was used to compare the goodness-of-fit between models, where lower DIC indicates a better model fit. An α-level of 0.05 was used in all analyses to indicate statistical significance (as indicated by 95% credible intervals (95% CrI) for relative risks (RR) that excluded 1).

Choropleth maps were created using the ArcGIS software [37] to visualise the geographical distribution of crude SMRs for the 227 townships in NHAR. The relative risks of infection were expressed as a percentage by multiplying by 100. The posterior means of the random effects obtained from the models were also mapped.

## Results

### Descriptive analysis

Summary statistics for annual mean numbers of human echinococcoses in NHAR for the period 1 January 1994–31 December 2013 were calculated (Table 2). A total of 4472 cases were diagnosed in the hospitals during the study period. From the total number of cases, 4402 cases (98.4%) were CE and 72 (1.6%) were AE. Two patients were diagnosed with both diseases. The number of annual cases of CE increased slightly from 1994 to 2013 (Additional file 7). Apart from the peak in the annual number of AE cases in 2007 and 2008, the annual human echinococcosis cases remained relatively stable during the study period (Additional file 8). While the number of annual CE cases by township ranged between 0 and 32 with a mean of 0.9, the annual number of AE cases ranged between 0 and 5 with a mean of 0.02. Annual maximum and minimum temperatures for the townships in NHAR were 26.3 °C and − 13.9 °C, respectively, with a mean of 8.7 °C between 1980 and 2013. In the same period, annual maximum precipitation was 19,981.3 mm and annual minimum precipitation was 0.01 mm with a mean of 255.6 mm (Additional files 9 and 10). The mean elevation of the administrative areas was 1506.3 m above sea level. Township area covered by each land cover class in NHAR for the period 1 January 1980 to 31 December 2013 is presented in Additional file 11.

**Table 2:** Numbers of total echinococcosis cases in Ningxia Hui Autonomous Region by year from 1994 to 2013

Year	Frequency total (CE/AE)	Percent of total cases (CE/AE) in the period (%)	Cumulative frequency total (CE/AE)	Cumulative percent total (CE/AE)
1994	141 (139/2)	3.2 (3.1/2.8)	141(139/2)	3.2 (3.1/2.8)
1995	208 (205/3)	4.7 (4.6/4.2)	349 (344/5)	7.8 (7.7/7.0)
1996	244 (240/4)	5.5 (5.4/5.5)	593 (584/9)	13.3 (13.1/12.5)
1997	270 (266/4)	6.0 (6.0/5.5)	863 (850/13)	19.3 (19.1/18.0
1998	244 (239/5)	5.5 (5.4/6.9)	1107 (1089/18)	24.8 (24.6/24.9)
1999	249 (243/6)	5.6 (5.5/8.3)	1356 (1332/24)	30.3 (30.1/33.2)
2000	275 (268/7)	6.1(6.1/9.7)	1631 (1600/31)	36.5 (36.2/42.9)
2001	195 (192/3)	4.4 (4.3/4.2)	1826 (1792/34)	40.8 (40.5/47.1)
2002	215 (214/2)	4.5 (4.8/2.8)	2041 (2006/36)	45.6 (45.3/49.9
2003	186 (184/2)	4.2 (4.2/2.8)	2227 (2190/38)	49.8 (49.5/52.7)
2004	213 (211/2)	4.8 (4.8/2.8)	2440 (2401/40)	54.6 (54.3/55.5)
2005	223 (221/2)	5.0 (5.0/2.8)	2663 (2622/42)	59.5 (59.3/58.3)
2006	189 (188/1)	4.2 (4.3/1.4)	2852 (2810/43)	63.8 (63.6/59.7)
2007	214 (201/13)	4.8 (4.6/18.1)	3066 (3011/56)	68.6 (68.2/77.8)
2008	255 (246/9)	5.7 (5.6/12.5)	3321(3257/65)	74.3 (73.8/90.3)
2009	283 (279/5)	6.3 (6.3/6.9)	3604 (3536/70)	80.6 (80.1/97.2)
2010	218 (218/0)	4.9 (4.9/0.0)	3822 (3754/70)	85.5 (85.0/97.2)
2011	205 (204/1)	4.6 (4.6/1.4)	4027 (3958/71)	90.0 (89.6/98.6)
2012	249 (249/0)	5.6 (5.6/0.0)	4276 (4207/71)	95.6 (95.2/98.6)
2013	196 (195/1)	4.4 (4.4/1.4)	4472 (4402/72)	100 (100/100)
Total	4472 (4402/72)	100 (100/100)	–	–

**Fig. 3:**
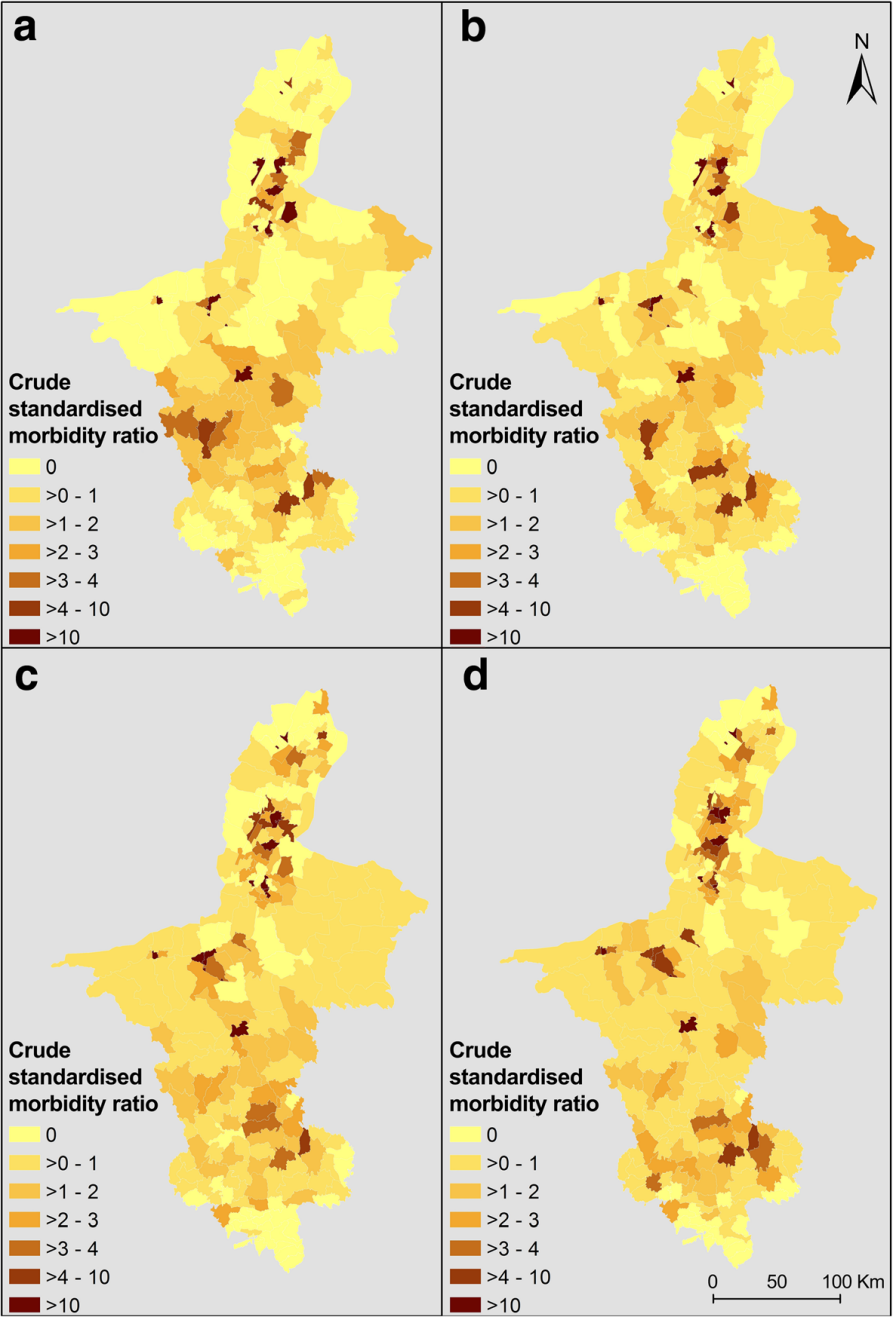
Raw standardised morbidity ratios for cystic echinococcosis by township in NHAR for four different periods: **a** 1994–1998; **b** 1999–2003; **c** 2004–2008; **d** 2009–2013

**Fig. 4:**
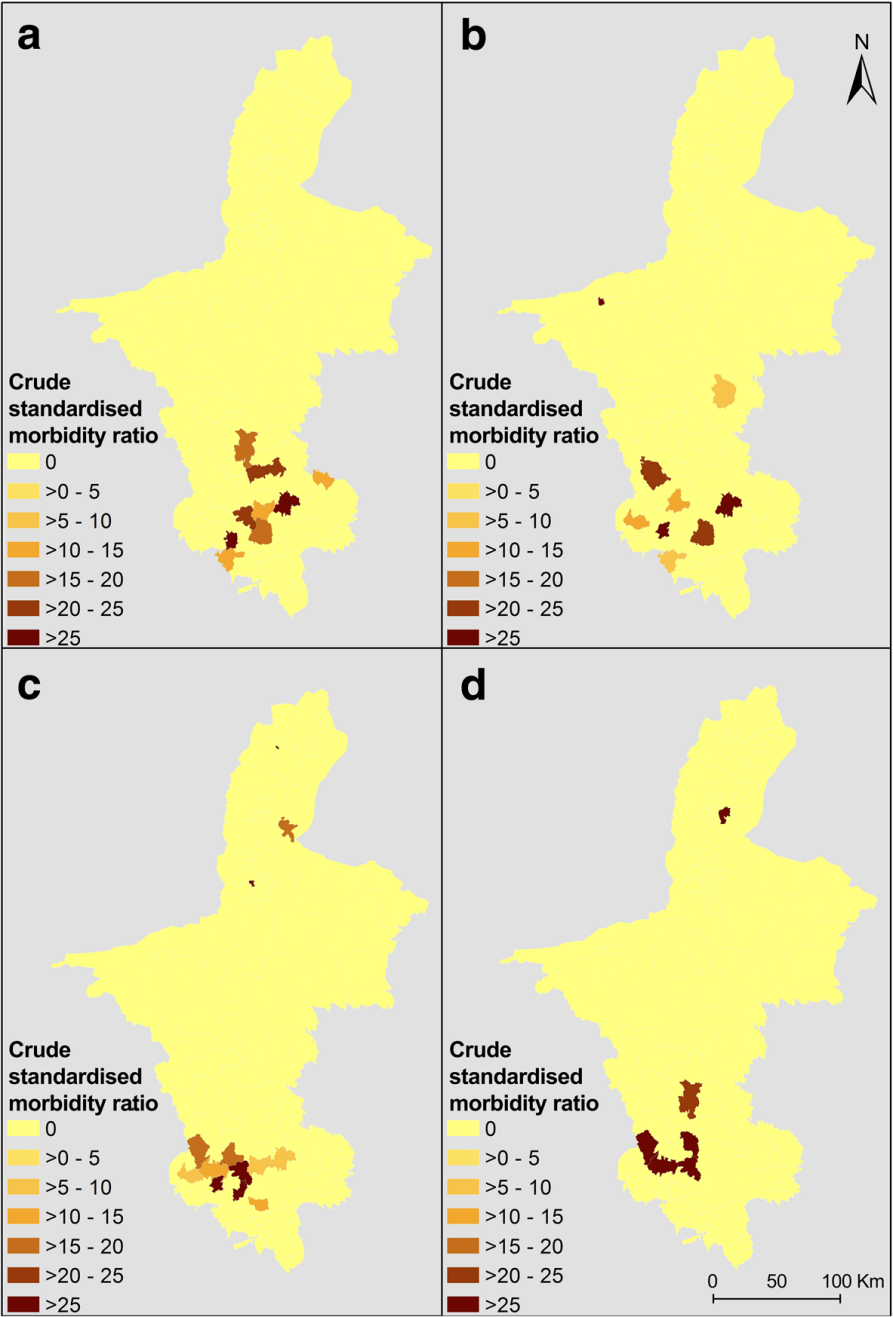
Raw standardised morbidity ratios for alveolar echinococcosis by township in NHAR for four different periods: **a** 1994–1998; **b** 1999–2003; **c** 2004–2008; **d** 2009–2013

The maps of SMRs for the number of CE infections by township in the four time periods show some degree of spatial variability across the province (Fig. 3). In general, higher incidence rates of CE were observed in townships located in the northern Yellow River Irrigated District and the southern mountainous and loess hilly district, whereas lower incidence rates were recorded in the central desertified district of NHAR. The maps of AE incidence show that this infection was mainly distributed in the South with occasional foci identified in the North (Fig. 4).

### Bayesian spatio-temporal models of human CE and AE

**Fig. 5:**
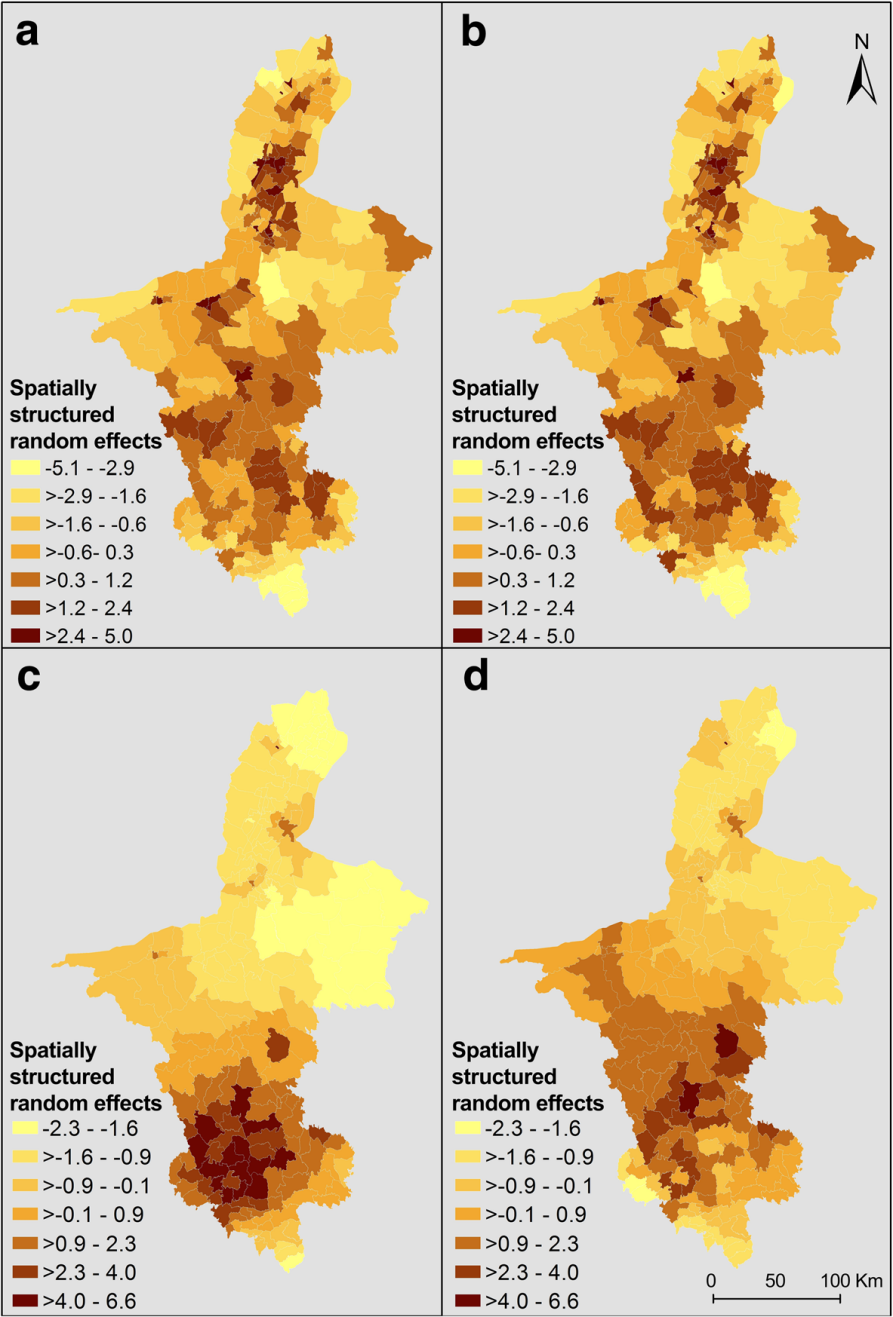
Spatial distribution of the posterior means of random effects for cystic and alveolar echinococcoses in NHAR. Spatially structured random effects of Models II (**a**) and III (**b**) of cystic echinococcosis, and spatially structured random effects of Models II (**c**) and III (**d**) of alveolar echinococcosis

Based on the DIC estimates, Models II of CE and AE had the best parsimonious characterization of the data among all the models examined (Tables 3, 4). The higher DIC for Model I and III than Model II indicates that the addition of spatial structure to the random effects improved model fit. In model II of CE, winter mean temperature at 10-year lag had a statistically significant association with the incidence of cases (Additional file 12). There was an estimated increase of 15.0% (95% CrI: 10.8–19.3%) in the risk of CE for a 1 °C increase in winter mean temperature 10 years prior to the diagnosis of the infection. Conversely, there was a decrease of 2.2% (95% CrI: 1.2–3.4%) in the risk of CE for every year during the study period. The quadratic term for annual mean temperature was also significant, indicating that the association between this variable and the outcome was nonlinear (Additional file 13). The MA2 of annual mean temperature, the MA4 of annual mean precipitation and the MAs calculated for the percentage of township area covered by the land cover types were not significant. The difference in the variance of the spatially structured random effect between Model III (9.1; 95% CrI: 7.4–11.6) and Model II (8.9; 95% CrI: 7.1–11.1) indicates that the covariates accounted for only a small proportion of the spatial variability in the data (Table 3 and Fig. 5a, b).

**Table 3:** Regression coefficients, RRs and 95% CrI from Bayesian spatial and non-spatial models for cystic echinococcosis in NHAR from 1 January 1994 to 31 December 2013

	Model I		Model II		Model II
Variable	Coefficient, posterior mean (95% CrI)	RRs, posterior mean (95% CrI)	Coefficient, posterior mean (95% CrI)	RRs, posterior mean (95% CrI)	Coefficient, posterior mean (95% CrI)
α (Intercept)	-0.38 (− 0.01– -0.38)	–	-0.40 (-0.56– -0.25)	–	-0.67 (-0.78– -0.60)
1. Annual mean temperature (°C) lag-13 years	0.04 (4.55 × 10^-4^–0.04)	1.04 (1.00–1.04)	0.04 (-0.03–0.11)	1.04 (0.97–1.12)	–
2. Annual mean temperature^2^ (°C) lag-13 years	-0.05 (-0.05–1.56 × 10^-4^)	0.95 (0.95–1.00)	-0.05 (-0.08– -0.02)	0.95 (0.92–0.98)	–
3. Winter mean temperature (°C) lag-10 years	0.14 (1.93 × 10^-4^–0.14)	1.15 (1.00–1.15)	0.14 (0.10–0.18)	1.15 (1.11–1.19)	–
4. Herbaceous vegetation lag-13 years	-0.01 (-0.01–7.08 × 10^-5^)	0.99 (0.99–1.00)	-0.01 (-0.01–2.86 × 10^-4^)	0.99 (0.98–1.00)	–
5. Bareland/artificial surfaces (%) MA5	0.01 (6.21 × 10^-5^–0.01)	1.01(1.00–1.01)	4.89 × 10^-3^ (-1.31 × 10^-3^–0.01)	1.00 (0.99–1.01)	–
6. Cultivated land (%) MA5	2.79 × 10^-3^ (5.02 × 10^-5^–2.79 × 10^-3^)	1.01 (1.00–1.01)	2.83 × 10^-3^ (-2.38 × 10^-3^–8.08 × 10^-3^)	1.00 (0.99–1.01)	–
7. Forest (%) MA5	4.09 × 10^-4^ (5.26 × 10^-5^–4.4 × 10^-4^)	1.01 (1.00–1.01)	5.52 × 10^-3^ (-3.93 × 10^-3^–0.01)	1.00 (0.99–.01)	–
8. Shrubland (%) MA5	-0.02 (-0.02–9.85 × 10^-5^)	0.98 (0.98–1.00)	-0.02 (-0.05–0.01)	0.98 (0.95–1.01)	–
9. Water bodies (%) MA6	-1.84 × 10^-3^ (-1.81 × 10^-3^–2.72 × 10^-5^)	0.99 (0.99–1.00)	-2.06 × 10^-3^ (-0.01–4.71 × 10^-3^)	0.99 (0.99–1.00)	–
10. Annual mean temperature (°C) MA2	-0.01 (-0.01–8.81 × 10^-5^)	0.99 (0.99–1.00)	-0.01 (-0.02–8.15 × 10^-3^)	0.99 (0.98–1.01)	–
11. Annual minimum precipitation (mm) MA4	-0.07 (-0.07–5.3 × 10^-4^)	0.93 (0.93–1.00)	-0.06 (-0.15–0.03)	0.94 (0.86–1.03)	–
12. Time (year)	-0.02 (-0.02–1.13 × 10^-4^)	0.98 (0.98–1.00)	-0.02 (-0.03– -0.01)	0.97 (0.96–0.98)	–
Heterogeneity unstructured	2.77 (2.79–2694.70)	–	–	–	–
Heterogeneity structured	–	–	8.98 (7.14–11.11)	–	9.14 (7.37–11.59)
DIC	9610	–	9396	–	9529

*Abbreviations*: *RRs* relative risks, *95% CrI* 95% credible interval

**Table 4:** Regression coefficients, RRs and 95% CrI from Bayesian spatial and non-spatial models for alveolar echinococcosis in NHAR from 1 January 1994 to 31 December 2013

	Model I		Model II		Model III
Variable	Coefficient, posterior mean (95% CrI)	RRs, posterior mean (95% CrI)	Coefficient, posterior mean (95% CrI)	RRs, posterior mean (95% CrI)	Coefficient, posterior mean (95% CrI)
α (Intercept)	-4.9 (-7.78– -2.81)	–	-5.33 (-7.97– -3.08)	–	-2.83 (-4.36– -1.76)
1. Bareland/artificial surfaces (%) MA6	-0.03 (-0.07–1.3 × 10^-3^)	0.97 (0.93–1.00)	-0.05 (-0.09– -0.01)	0.95 (0.91–0.99)	–
2. Forest (%) MA4	-0.04 (-0.11–0.03)	0.96 (0.89–1.03)	-0.06 (-0.14–0.01)	0.94 (0.87–1.01)	–
3. Winter mean precipitation (mm) MA1	-0.01 (-0.05–0.02)	0.99 (0.95–1.02)	-0.01 (-0.06–0.02)	0.99 (0.94–1.02)	–
4. Annual mean temperature (°C) MA2	1.18 (-0.31–2.65)	3.26 (0.73–14.18)	1.33 (-0.23–2.91)	3.79 (0.80–18.45)	–
5. Annual mean temperature (°C) MA6	-3.63 (-6.07– -1.36)	0.02 (2.31 × 10^-3^–0.26)	-3.63 (-6.20– -1.23)	0.03 (2.03 × 10^-3^–0.29)	–
6. Annual temperature range (°C) MA2	-0.34 (-1.04–0.40)	0.71 (0.35–1.49)	-0.20 (-0.98–0.60)	0.82 (0.37–1.81)	–
7. Winter mean temperature (°C) MA1	-1.03 (-1.86– -0.18)	0.36 (0.16–0.84)	-1.07 (-1.92– -0.22)	0.34 (0.15–0.80)	–
8. Winter mean temperature (°C) MA6	0.42 (-0.68–1.51)	1.52 (0.51–4.52)	0.32 (-0.92–1.53)	1.38 (0.40–4.62)	–
9. Winter mean temperature^2^ (°C) MA6	-0.47 (-1.14–0.11)	0.62 (0.32–1.12)	-0.50 (-1.18–0.11)	0.61 (0.30–1.12)	–
10. Time (year)	0.17 (0.05–0.30)	1.20 (1.06–1.35)	0.18 (0.05–0.32)	1.20 (1.05–1.37)	–
Heterogeneity unstructured	3.51 (1.66–9.27)	–	–	–	–
Heterogeneity structured	–	–	9.47 (4.60–23.82)	–	10.63 (5.54–25.01)
DIC	485.2	–	184.8	–	486.7

Model II of AE showed that the MA1 of winter mean temperature (Additional file 14), the MA6s of annual mean temperature (Additional file 15) and the percentage of township area covered by bareland/artificial surfaces, had a significant negative association with AE cases. There was a decrease of 65.7% (95% CrI: 19.6–85.4%) in the risk of AE for a 1 °C increase in the average of winter temperature calculated for the 5-year period previous to the diagnosis of the disease (0–4 years). Also, the decrease in the risk of AE was 97.4% (95% CrI: 70.8–99.8%) and 5.0% (95% CrI: 0.9–9.3%) for an increase of 1 °C in annual mean temperature and 1% increase in MA6 of township area covered by bareland/artificial surfaces, respectively. There was a statistically significant increasing temporal trend in the risk of AE. The difference between the DIC of Model II, 184.8, and that of model III, 486.7, indicates that the inclusion of the environmental covariates improved model parsimony. The variance of the spatially structured random effect decreased from 10.6 (95% CrI: 5.5–25.0) in Model III to 9.5 (95% CrI: 4.6–23.8) in Model II. These results may suggest that, unlike the findings in the model of CE, the selected environmental covariates characterised a higher proportion of the spatial variation in the risk of AE (Fig. 5c, d).

The maps of the residual spatial variation of CE, before (Model III) and after (Model II) accounting for the environmental covariates, show almost identical spatial patterns without clear evidence of disease clustering (Fig. 5a, b). Conversely, the maps of the distribution of the residual spatial variation of AE risk demonstrated evidence of clustering when the model did not incorporate the environmental covariates (Model III). The degree of clustering decreased when the effect of these variables was included (Model II), suggesting that the covariates contributed to clustering in the south of NHAR (Fig. 5c, d). Maps of the raw relative risks were generated for CE and AE by township and year (Additional files 16 and 17). These maps show that the risk of CE was distributed across all geographic regions in NHAR during the entire study period, while the risk of AE was confined to the south. However, based on the environmental factors associated with AE risk in NHAR, it was also possible to identify an area at high risk of AE in the northeastern part of the central desertified district (Additional file 17).

## Discussion

The results indicate that winter mean temperature and annual mean temperature, 10 and 13 years prior to diagnosis, respectively, have influenced the incidence of *E. granulosus* at the township level in NHAR. Temperature is a major determinant of the survival and longevity of *Echinococcus* spp. eggs in the external environment [59, 60]. In vivo studies have concluded that the eggs of *E. granulosus* remain viable and infective after 41 months of exposure to an inferior arid climate, which is characterised by large thermal amplitude (from − 3 to 37 °C) and low precipitation (under 300 mm/year) [59]. The present study revealed a positive association of CE cases with winter temperature at 10-year lag and a non-linear association with annual mean temperature at 13-year lag. These findings indicate that the number of CE cases may have increased progressively when eggs were exposed to optimal temperatures but decreased with extreme temperatures that fell outside the optimal range. The relationship between *E. granulosus* infection and these two variables was significant after a time lag of more than 10 years. This is in agreement with the long incubation period of this parasite that has been reported to be between 5 and 15 years [41]. Of note, we do not suggest that the specific lag periods for each variable are important, but that the general pattern of lags indicate environmental conditions in the range of 10 to 15 years previously influence current patterns of disease.

CE cases were distributed across all the three biogeographical areas of NHAR: the northern Yellow River Irrigated District, the central desertified district and the southern mountainous and loess hilly district (Fig. 1). A higher risk of infection was observed in townships located in the North in close geographical proximity to Yinchuan. Urban areas may provide better job prospects and higher salaries for rural migrants who were exposed in their home township. In the cities, people who contracted the infection in their rural areas of origin may have had an improved access to healthcare services and the confirmation of the diagnosis of echinococcosis and management [61]. These findings raise the need for further studies to determine how access to healthcare may affect the incidence of the infection. The risk of CE was found to be higher in townships from the southern mountainous and loess hill district. This part of NHAR is one of the three poorest areas in China where almost half the population belong to the Hui minority ethnic group [16]. Livestock and arable farming, which are common practices among these communities, represent higher risk of exposure to *Echinococcus* spp. [62, 63]. The Provincial technical standards for livestock slaughtering and antemortem and *post-mortem* meat inspection in NHAR are in agreement with the recommendations proposed by Food and Agriculture Organization of the United Nations [64, 65]. However, government-run abattoirs in NHAR are scarce, particularly in the South, where livestock slaughter is carried out mostly at rural market places or in domestic environments that are not legally compliant [66]. Unrestricted post-slaughter offal disposal is common in the region and has been identified as a potential local factor increasing the risk of CE [67]. Under similar circumstances in Qinghai Province, previous studies have suggested that domestic dogs may have a higher probability of access to livestock viscera in early winter and spring [68]. The prevalence of CE in sheep was estimated to be 52% in NHAR in 2008, and between 0 and 9% according to more recent reports of studies conducted at small spatial scale (no larger than county level) [66, 69, 70]. The variance in these prevalence estimates may be related to local or individual conditions that favour hotspots of high transmission within discrete patches of CE endemicity. Also, in the Autonomous Region, 3% of goats, 81% of cattle, 24% of pigs and 19% of camels were reported infected with *E. granulosus* in 2008 [71]. Although there is evidence of spatial clustering within the central desertified district, lower risk of CE was observed in this biogeographical area where communities are more scattered in isolated settlements.

The environmental covariates accounted for a relatively small proportion of the spatiotemporal variation in CE risk in NHAR. These findings suggest that there may be other local unmeasured factors that influence the spatial distribution of *E. granulosus* in the province. Some local socio-economic and behavioural drivers that have also been found to be positively related to CE in this hyperendemic area include low income, limited education, poor hygiene practices, female gender and Hui ethnicity. In contrast, tap water consumption has been identified as a factor that can protect against *E. granulosus* infection [35]. Although infection control in dogs has been identified as a key measure against echinococcoses in China, dog ownership still remains as an important risk factor for the infection in NHAR [35, 72]. The western China echinococcosis control programme recommends supervised treatment of all owned dogs four to eight times a year with praziquantel [73]. However, this is a measure that has been hard to apply, monitor and sustain in the remote-settled communities of the Autonomous Region [74].

The findings of the model of AE concur with previous studies conducted in different regions in echinococcosis-endemic countries that indicated that *E. multilocularis* has a focal spatial distribution [6–8]. The study also concurs with previous evidence that indicates that land cover and temperature influence AE incidence [22, 60, 75]. AE risk was spatially clustered in an area covered by Xiji, Guyuan and Haiyuan Counties, located in the southern mountainous and loess hill district (Fig. 1). This part of NHAR has been greatly transformed by the implementation of the GGP. Forest growth has primarily occurred in the northern and southern parts, in the Helan and Liupan mountains in the North and South, respectively (Fig. 1) [76, 77]. An increase in herbaceous vegetation has also been described in the central arid area of NHAR, and around the margin of the forestland [76, 77]. Hence, the distribution of AE risk observed in the current study concurs with the spatial patterns of the GGP land conversion processes that have been described in this autonomous region.

*Echinococcus multilocularis* is transmitted in semi-domestic and sylvatic life cycles that involve dogs and foxes as main definitive hosts, respectively, and small mammals as intermediate hosts [6, 78]. It has been demonstrated that landscape structure may influence the transmission patterns of this parasite by influencing the suitability and location of ecological habitats for its hosts [11]. With regards to land cover, it was found that the merged category of bareland/artificial surfaces was not associated with the transmission of *E. multilocularis* at the township level in NHAR. This observation suggests that the life-cycle of the parasite is supported in vegetated areas (i.e. forest, shrubland and cropland). These findings raise the need for further studies to determine the association of the GGP and other potential drivers of land cover change with the risk of human AE.

The impact of forest fragmentation on small mammals assemblages has now been demonstrated and explained by the interaction between forest patch metrics and small mammal species richness, abundance and composition [21, 31, 79–81]. In Xiji County, in the South of NHAR, a previous study indicated that the abundance of degraded lowland pasture was related to higher prevalence of AE in humans [14]. In the same area, a small-mammal survey conducted in relation to different land cover types at the community level revealed the presence of 16 species of small mammals [11]. That study indicated that in areas that experienced afforestation, the diversity of assemblages was lower than that of assemblages in areas where deforestation occurred [11]. However, species richness did not seem to be affected by these land conversion processes [11]. Trapping success of potential *E. multilocularis* intermediate hosts such as, *Cricetulus longicaudatus* and *Ochotona daurica*, was higher in recently afforested set-aside fields and abandoned grasslands, and *Spermophilus alashanicus*/*dauricus* in young plantations [11]. Therefore, it can be assumed that landscape transformation process that is taking place in NHAR may have disturbed rodent assemblages, and therefore affect the risk of *E. multilocularis* transmission. In Zhang County, Gansu Province, a study revealed that the process of land cover change that occurred in this endemic area affected the transmission dynamics of the parasite. There, the increase in grassland/shrubland that followed a process of deforestation favoured the creation of peri-domestic habitats of intermediate host species, and the development of peri-domestic cycles involving dogs [13, 82]. Similarly, the percentage of area covered by grassland and *E. multilocularis* infection in humans and foxes had a positive relationship in Eastern France [13, 83, 84]. In this area various studies also reported regular outbreaks of *Microtus arvalis* and *Arvicola terrestris*, key intermediate hosts for *E. multilocularis* [13, 17]. However, the picture is complex, given that in Sichuan Province, a negative cross-sectional association was observed between *Ochonta* spp. and Enhanced Vegetation Index, and previous evidence showed that this recognised intermediate host of *E. multilocularis* is more common in areas with low vegetation cover [16, 85, 86].

The negative association between AE cases and winter temperatures may be due to the influence of temperature exposure on eggs of *E. multilocularis*, and potentially the influence of temperature on small mammal population dynamics and fox/dog/small mammal predator-prey relationships [60, 87]. Evidence indicates that temperature affects the geographical range and changes the composition of small mammal communities [88, 89]. Also, climate has been identified as a factor contributing to changes in the distribution and abundance of the red and Arctic foxes, which are sylvatic definitive hosts for *E. multilocularis* in Arctic Canada [90, 91]. Reports of infection with *E. multilocularis* in red foxes in NHAR are only available for the mid-1980s [92]. At that time, 15% of trapped red foxes were infected with *E. multilocularis* in Xiji and Guyuan Counties [92]. Although there is not current evidence on how the local environment fluctuations influence the ecology of this type of vertebrates in the Autonomous Region, it can be thought that variations in climate and land cover have the potential to affect the dynamics and predator-prey interactions of potential hosts for *E. multilocularis* in NHAR. Also, climate and the landscape may favour the presence of other potential definitive hosts for this parasite in NHAR. Infection with *E. multilocularis* has also been detected in wolves (*Canis lupus*) and corsac foxes (*Vulpes corsac*) in other parts of the P.R. China [82].

Since the latent phase of AE in humans has been estimated to be between 5 and 15 years, the associations between AE incidence and land cover and temperature are plausible [93]. However, in this study there was also a significant association between AE cases with the average of winter temperature for the years 0, 1, 2, 3 and 4 prior to diagnosis. This finding may suggest other effects of temperature on the health-seeking behaviour of the inhabitants of NHAR, rather than on exposure to, or infection with the parasite. The high cost of medical care and the lack of health insurance have been identified previously as primary factors for the under-utilization of health care services in China [94, 95]. Therefore, seasonal rural-urban migration and temporary employment in NHAR could be related to this association between winter temperature and the risk of human AE.

As initiatives to address the high burden of human echinococcoses in China have already been established [27], there is a current need to identify high-risk areas for undetected infection to provide guidance to local authorities in implementation of the surveillance and control interventions [27]. The present study provides important evidence that indicates that populations living in southern mountainous and loess hilly district of NHAR were at greatest risk of acquiring CE or AE during the study period. Hence, these findings can be used to inform a decision to prioritise screening surveys in communities from Xiji, Guyuan and Haiyuan Counties which areas heavily affected by both forms of the infection. In this way, it will be possible to provide better estimates of the real impact of human echinococcoses in the autonomous region and to monitor the patterns of *E. granulosus* and *E. multilocularis* transmission [96]. To further improve the predictive performance of our models, particularly in remote areas with limited access to health services, the surveillance data should be analysed with other socio-demographic data [18]. The use of GIS technologies, Earth observation data and spatial statistical analysis for the study of the spatio-temporal dynamics of CE and AE cases may help to monitor trends in echinococcosis occurrence in hyperendemic regions. This information is relevant particularly in areas where ecological projects that alter local ecosystems are currently being implemented. Therefore, these technologies may be used to estimate future requirements for scaling up and targeting of essential control strategies, and to provide risk assessments for future landscape planning and ecosystem management and protection initiatives [19].

The limitations of this study include that it relied mainly on data collected from selected county hospitals, which overlooks the contribution of CE and AE cases that were not referred to these health care institutions for confirmation of diagnosis treatment and follow-up. Human echinococcoses are not legally notifiable diseases in China. Most patients are commonly identified in clinical records and mass screening surveys conducted in the most affected areas to help reduce the medical, social and economic burden of the infections. Therefore, further work needs to be carried out to evaluate and improve the surveillance and provide real estimates of the number echinococcosis cases in the country. Also, in this study, data on the number of patients who were immunosuppressed at the time of diagnosis were not available. Among these patients, CE and AE behave differently and may develop during a relatively short period of time [97]. Therefore, it is recommended that future studies to identify environmental risk factors for transmission also involve indices of individual biological condition that may be associated with progression and times of infection and diagnosis of the disease. In the study, the township in which patients resided at the time of diagnosis was assumed as the place where acquisition of infection occurred. Although the patient’s place of residence may be a reliable indicator for establishing the geographical origin of the infections, this may not apply for all cases. The human labour migration that has characterised NHAR in past decades may have had an impact on the observed trends of infection and results need to be interpreted with caution. Here, we explored the spatio-temporal patterns of echinococcosis infection in NHAR, and the association of environmental variables with the transmission of *Echinococcus* spp. at the township level. Hence, the results do not allow for inferences to be made about the risk of human echinococcoses at the commune or individual levels. More detailed information about the local structure of these infections may be further included to improve the CE and AE models. The impact of the GGP and other ecological restoration projects was not formally tested in this study. Therefore, it is necessary to establish evidence for the impact of such projects to facilitate environmental risk assessments of future ecosystem management and protection programmes. [98]. The use of interpolated surfaces for the estimation of climatic and land cover variables also represented a challenge for the interpretation of the findings. The precision of the interpolated values at point locations may vary considerably over time and over the entire study area. Also, the IDW interpolation method used by the Chinese Academy of Sciences is a simple and intuitive deterministic method based on the assumption that sample values closer to the prediction location have more influence on prediction value than sample values farther apart. However, IDW has sensitivity to outliers or sampling configuration and does not allow the incorporation of ancillary data [99, 100]. We believe that a meaningful assessment of the associations between human echinococcosis risk and the environment can only be achieved with the use of consistent and long-term climate and land cover records that allow to capture significant spatial variability.

## Conclusions

In this study, maps of the geographical distribution of CE and AE for a highly endemic area of China (NHAR) have been produced, and some of the environmental factors that are associated with the transmission patterns of *E. granulosus* and *E. multilocularis* at the township level were quantified. Selected environmental covariates characterised a large proportion of the spatiotemporal variation in the risk of AE. However, the CE appears to be less spatially variable and the geographical distribution is likely determined by other unmeasured factors. Evidence on the potential effects of the GGP on the risk of AE was presented due to the association with vegetated areas and a decrease in bareland. By mapping the distribution of the risk of these infections, we provide evidence that can be used to scale-up and target essential control strategies, and to inform risk assessment of large-scale landscape regeneration initiatives.

## Additional files

Additional file 1: Percent population change in NHAR for the periods 1980–1990, 1991–2001 and 2002–2013. (JPEG 3042 kb)

Additional file 2: Spatial distribution of the average annual mean temperature in °C in NHAR for the period 1980–2013. (DOCX 209 kb)

Additional file 3: Spatial distribution of the average annual mean precipitation in mm in NHAR for the period 1980–2013. (DOCX 182 kb)

Additional file 4: Maps of the spatial distribution of **a** annual, **b** summer and **c** winter temperature trends, and **d** annual, **e** summer and **f** winter precipitation trends in NHAR for the period 1 January 1980 to 31 December 2013. Note, the values presented in the figure are relative to the provincial average per decade. (DOCX 244 kb)

Additional file 5: OpenBUGS code used to develop the Bayesian spatial model (Model II) for cystic echinococcosis in NHAR from 1 January 1994 to 31 December 2013. (DOCX 13 kb)

Additional file 6: OpenBUGS code used to develop the Bayesian spatial model (Model II) for alveolar echinococcosis in NHAR from 1 January 1994 to 31 December 2013. (DOCX 13 kb)

Additional file 7: Number of observed and expected number of CE cases by year (1994–2013) in NHAR for the period 1 January 1994 to 31 December 2013. (DOCX 78 kb)

Additional file 8: Number of observed and expected number of AE cases by year (1994–2013) in NHAR for the period 1 January 1994 to 31 December 2013. (DOCX 72 kb)

Additional file 9: Annual temperature in NHAR for the period 1 January 1980 to 31 December 2013 and number of cases of CE and AE for the period 1 January 1994 to 31 December 2013. (DOCX 98 kb)

Additional file 10: Annual precipitation in NHAR for the period 1 January 1980 to 31 December 2013 and number of cases of CE and AE for the period 1 January 1994 to 31 December 2013. (DOCX 119 kb)

Additional file 11: Township area covered by each land cover class in NHAR for the period 1 January 1980 to 31 December 2013 and number of cases of CE and AE for the period 1 January 1994 to 31 December 2013. (DOCX 208 kb)

Additional file 12: Scatterplots of number of CE cases by township against winter mean temperature at a 10-year lag. (DOCX 135 kb)

Additional file 13: Scatterplots of number of CE cases by township against annual mean temperature at 13-year lag. (DOCX 145 kb)

Additional file 14: Scatterplots of number of AE cases by township against winter mean temperature for the period 0–4 years before diagnosis. (DOCX 75 kb)

Additional file 15: Scatterplots of number of AE cases by township against annual mean temperature calculated for the period 11–15 years before diagnosis. (DOCX 72 kb)

Additional file 16: Spatial distribution of annual raw relative risks for CE in NHAR for the period 1994 to 2013. (JPEG 7859 kb)

Additional file 17: Spatial distribution of annual relative risks for AE in NHAR for the period 1994 to 2013. (JPEG 7722 kb)

## References

[1] McManus DP, Gray DJ, Zhang W, Yang Y (2012). Diagnosis, treatment, and management of echinococcosis. BMJ.

[2] Eckert J, Deplazes P (2004). Biological, epidemiological, and clinical aspects of echinococcosis, a zoonosis of increasing concern. Clin Microbiol Rev.

[3] McManus DP, Zhang W, Li J, Bartley PB (2003). Echinococcosis. Lancet.

[4] Moro P, Schantz PM (2009). Echinococcosis: a review. Int J Infect Dis.

[5] Ministry of Health (2008). Report on the national survey of current status of major human parasitic diseases in China.

[6] Eckert J (2001). WHO/OIE manual on echinococcosis in humans and animals: a public health problem of global concern.

[7] Giraudoux P, Pleydell D, Raoul F, Quéré J-P, Wang Q, Yang Y, et al. Transmission ecology of *Echinococcus multilocularis*: what are the ranges of parasite stability among various host communities in China? Parasitol Int. 2006;55:S237–46. 10.1016/j.parint.2005.11.036 16361111

[8] Giraudoux P, Raoul F, Afonso EVE, Ziadinov I, Yang Y, Li LI, et al. Transmission ecosystems of *Echinococcus multilocularis* in China and central Asia. Parasitology. 2013;140(13):1655–66. 10.1017/S0031182013000644 PMC3806041 23734823

[9] Sen-Hai Y, Hu W, Xian-Hong W, Xiao M, Pei-Yun L, Yu-fang L (2008). Cystic and alveolar echinococcosis: an epidemiological survey in a Tibetan population in southeast Qinghai, China. Jpn J Infect Dis.

[10] Zhang W, Zhang Z, Wu W, Shi B, Li J, Zhou X (2015). Epidemiology and control of echinococcosis in central Asia, with particular reference to the People's Republic of China. Acta Trop.

[11] Raoul F, Pleydell D, Quéré J-P, Vaniscotte A, Rieffel D, Takahashi K (2008). Small-mammal assemblage response to deforestation and afforestation in central China. Mammalia.

[12] Raoul F, Deplazes P, Rieffel D, Lambert J-C, Giraudoux P (2010). Predator dietary response to prey density variation and consequences for cestode transmission. Oecologia.

[13] Giraudoux P, Craig P, Delattre P, Bao G, Bartholomot B, Harraga S (2003). Interactions between landscape changes and host communities can regulate *Echinococcus multilocularis* transmission. Parasitology.

[14] Pleydell DR, Yang YR, Danson FM, Raoul F, Craig PS, McManus DP (2008). Landscape composition and spatial prediction of alveolar echinococcosis in southern Ningxia, China. PLoS Negl Trop Dis.

[15] Wang Q, Vuitton DA, Xiao Y, Budke CM, Campos-Ponce M, Schantz PM (2006). Pasture types and *Echinococcus multilocularis*, Tibetan communities. Emerg Infect Dis.

[16] The Ecosystems Services for Poverty Alleviation (ESPA) Programme (2008). China ecosystem services and poverty alleviation situation analysis and research strategy. Ningxia case study.

[17] Giraudoux P, Delattre P, Habert M, Quéré JP, Deblay S, Defaut R (1997). Population dynamics of fossorial water vole (*Arvicola terrestris* Scherman): a land use and landscape perspective. Agric Ecosyst Environ.

[18] Atkinson J-AM, Gray DJ, Clements ACA, Barnes TS, McManus DP, Yang YR (2013). Environmental changes impacting *Echinococcus* transmission: research to support predictive surveillance and control. Glob Chang Biol.

[19] Cadavid Restrepo AM, Yang Y, McManus D, Gray D, Giraudoux P, Barnes T (2015). The landscape epidemiology of echinococcoses. Infect Dis Poverty..

[20] Liu C, Wu B (2010). Grain for Green Programme in China: Policy making and implementation. Policy Briefing Series.

[21] Vieira MV, Olifiers N, Delciellos AC, Antunes VZ, Bernardo LR, Grelle CE (2009). Land use vs. fragment size and isolation as determinants of small mammal composition and richness in Atlantic Forest remnants. Biol Conserv.

[22] Giraudoux P, Quéré J-P, Delattre P, Bao G, Wang X, Shi D (1998). Distribution of small mammals along a deforestation gradient in southern Gansu, central China. Acta Theriol (Warsz).

[23] Raoul F, Quéré J-P, Rieffel D, Bernard N, Takahashi K, Scheifler R (2006). Distribution of small mammals in a pastoral landscape of the Tibetan plateaus (western Sichuan, China) and relationship with grazing practices. Mammalia.

[24] Wang Q, Vuitton DA, Qiu J, Giraudoux P, Xiao Y, Schantz PM (2004). Fenced pasture: a possible risk factor for human alveolar echinococcosis in Tibetan pastoralist communities of Sichuan, China. Acta Trop.

[25] Wang Q, Raoul F, Budke C, Craig PS, Xiao Y-F, Vuitton DA (2010). Grass height and transmission ecology of *Echinococcus multilocularis* in Tibetan communities, China. Chin Med J.

[26] Yang G-J, Liu L, Zhu H-R, Griffiths SM, Tanner M, Bergquist R (2014). China's sustained drive to eliminate neglected tropical diseases. Lancet Infect Dis.

[27] Ministry of Health of the People's Republic of China (2006). National control program of key parasitic diseases in 2006–2015.

[28] Wang Q, Huang Y, Huang L, Yu W, He W, Zhong B (2014). Review of risk factors for human echinococcosis prevalence on the Qinghai-Tibet plateau, China: a prospective for control options. Infect Dis Poverty.

[29] National Bureau of Statistics. Population data. 2016. http://data.stats.gov.cn/english/easyquery.htm?cn=C01.

[30] United Nations Educational, Scientific and Cultural Organization. Migrant/Migration. 2017. http://www.unesco.org/new/en/social-and-human-sciences/themes/international-migration/glossary/migrant/. Accessed 2 Oct 2017.

[31] Du Y, Park A, Wang S (2005). Migration and rural poverty in China. J Comp Econ.

[32] Démurger S, Wan H (2012). Payments for ecological restoration and internal migration in China: the sloping land conversion program in Ningxia. IZA J Migr.

[33] Zhao Z (2005). Migration, labor market flexibility, and wage determination in China: a review. Dev Econ.

[34] Yang YR, Sun T, Li Z, Li X, Zhao R, Cheng L (2005). Echinococcosis, Ningxia, China. Emerg Infect Dis.

[35] Yang YR, Sun T, Li Z, Zhang J, Teng J, Liu X (2006). Community surveys and risk factor analysis of human alveolar and cystic echinococcosis in Ningxia hui autonomous region, China. Bull World Health Organ.

[36] The WorldPop population mapping program. The WorldPop Project. 2017. http://www.worldpop.org.uk/. Accessed 21 Oct 2015.

[37] ESRI: Environmental Systems Research Institute. ArcGIS Software version 10.3.1. http://www.esri.com/arcgis/about-arcgis.

[38] National Bureau of Statistiscs of China (2014). The year book-Population.

[39] Wachter KW (2014). Essential demographic methods.

[40] Malthus TR (1809). An essay on the principle of population, as it affects the future improvement of society. Vol. 2..

[41] Ammann RW, Eckert J. Cestodes. *Echinococcus*. Gastroenterol Clin North Am. 1996;25(3):655–89. 10.1016/s0889-8553(05)70268-5 8863045

[42] The United States Geological Survey (USGS). EarthExplorer. 2016. http://earthexplorer.usgs.gov/. Accessed 21 Oct 2016.

[43] Department of the Interior - The United States Geological Survey (USGS). Landsat 4–7 Climate Data Record (CDR) Surface Reflectance, Version 6.4. Product Guide. 2016. https://landsat.usgs.gov/landsat-surface-reflectance-data-products. Accessed 2 May 2016.

[44] Department of the Interior - The United States Geological Survey (USGS). Provisional Landsat 8 Surface Reflectance Product. https://landsat.usgs.gov/landsat-surface-reflectance-data-products. Accessed 2 May 2016.

[45] R Core Team (2015). R. A language and environment for statistical computing.

[46] Goslee SC (2011). Analyzing Remote sensing data in R: the landsat package. J Stat Softw.

[47] Exelis Visual Information Solutions I. ENVI software version 5.3. http://www.harrisgeospatial.com/ProductsandSolutions/GeospatialProducts/ENVI.aspx.

[48] National Geomatics Center of China. GlobeLand30. A 30-meter Global Land Cover Dataset. 2010. http://www.globallandcover.com/user/login.aspx?para=1. Accessed 12 Dec 2015.

[49] Global 25m Resolution PALSAR-2/PALSAR Mosaic and Forest/Non-Forest Map. 2010. http://www.eorc.jaxa.jp/ALOS/en/palsar_fnf/fnf_index.htm. Accessed 12 Nov 2015.

[50] Google Inc. Panoramio. Photos of the world 2005. https://www.panoramio.com/. Accessed 15 June 2015.

[51] Fonte CC, Bastin L, See L, Foody G, Lupia F (2015). Usability of VGI for validation of land cover maps. Int J Geogr Inf Sci.

[52] Google Earth Pro version 7.1.5.1557. https://www.google.com.au/earth/. Accessed 15 June 2015.

[53] Cadavid Restrepo AM, Yang YR, Hamm NA, Gray DJ, Barnes TS, Williams GM (2017). Land cover change during a period of extensive landscape restoration in Ningxia hui autonomous region, China. Sci Total Environ.

[54] The National Aeronautics and Space Administration (NASA) and Ministry of Economy Trade and Industry (METI). The Advanced Spaceborne Thermal Emission and Reflection Radiometer (ASTER) Global Digital Elevation Model (GDEM). Version 2. ASTER GDEM is a product of NASA and METI. 2011. https://asterweb.jpl.nasa.gov/gdem.asp. Accessed 16 Nov 2015.

[55] Pitney Bowes Software Inc. MapInfo, Group 1 Software and MapInfo Professional. https://www.pitneybowes.com/us/location-intelligence/geographic-information-systems/mapinfo-pro.html.

[56] Vuong QH. Likelihood ratio tests for model selection and non-nested hypotheses. Econometrica. 1989:307–33.

[57] Members of OpenBUGS Project Management Group. OpenBUGS software version 3.2.2 rev 1012. 2014. http://www.openbugs.net/w/Downloads.

[58] Besag J, York J, Mollié A (1991). Bayesian image restoration, with two applications in spatial statistics. Ann Inst Stat Math.

[59] Thevenet PS, Jensen O, Drut R, Cerrone GE, Grenóvero MS, Alvarez HM (2005). Viability and infectiousness of eggs of *Echinococcus granulosus* aged under natural conditions of inferior arid climate. Vet Parasitol.

[60] Veit P, Bilger B, Schad V, Schäfer J, Frank W, Lucius R (1995). Influence of environmental factors on the infectivity of *Echinococcus multilocularis* eggs. Parasitology.

[61] Hesketh T, Jun YX, Lu L, Mei WH (2008). Health status and access to health care of migrant workers in China. Public Health Rep.

[62] Cringoli G, Rinaldi L, Musella V, Veneziano V, Maurelli MP, Di Pietro F (2007). Geo-referencing livestock farms as tool for studying cystic echinococcosis epidemiology in cattle and water buffaloes from southern Italy. Geospat Health.

[63] Carmona C, Perdomo R, Carbo A, Alvarez C, Monti J, Grauert R (1998). Risk factors associated with human cystic echinococcosis in Florida, Uruguay: results of a mass screening study using ultrasound and serology. Am J Trop Med Hyg..

[64] Food and Agriculture Organization of the United Nations. Manual on meat inspection for developing countries. 1994. http://www.fao.org/docrep/003/t0756e/T0756E00.htm. Accessed 21 June 2017.

[65] Food and Agriculture Organization of the United Nations. Guidelines for humane handling, transport and slaughter of livestock. 2001. http://www.fao.org/3/a-x6909e.pdf. Accessed 21 June 2017.

[66] Cleary E, Barnes TS, Xu Y, Zhao H, Clements AC, Gray DJ (2014). Impact of “grain to green” Programme on echinococcosis infection in Ningxia hui autonomous region of China. Vet Parasitol.

[67] Yang YR, Clements AC, Gray DJ, Atkinson J-AM, Williams GM, Barnes TS, McManus DP. Impact of anthropogenic and natural environmental changes on *Echinococcus* transmission in Ningxia hui autonomous region, the People’s Republic of China. Parasit Vectors. 2012;5:146. 10.1186/1756-3305-5-146 PMC3419675 22827890

[68] Wang H, Ma S, Cao D, Zhao H, Liu F, Schantz P, et al. An epidemiological survey on human hydatidosis in southern Qinghai Plateau. Chin J Parasit Dis Con. 2000;13(1):37–41.

[69] Ma T, Wu X, Marvin F, Yun L. Infection prevalence study of the main hosts of *Echinococcus* in Ningxia. Ning Med J. 2014;4:376e378.

[70] Wu X (2015). Investigation of Ningxia livestock hydatid infection status in 2012. Ning Med J.

[71] Zhenghuan W, Xiaoming W, Xiaoqing L (2008). Echinococcosis in China, a review of the epidemiology of *Echinococcus* spp. EcoHealth.

[72] Yang YR, Cheng L, Yang SK, Pan X, Sun T, Li X, et al. A hospital-based retrospective survey of human cystic and alveolar echinococcosis in Ningxia Hui Autonomous Region, PR China. Acta Trop. 2006;97(3):284–91. 10.1016/j.actatropica.2005.12.001 16414005

[73] World Health Organization and World Organisation for Animal Health. Report of the WHO informal working group on cystic and alveolar echinococcosis surveillance, prevention and control, with the participation of the Food and Agriculture Organization of the United Nations and the World Organisation for Animal Health. 2011. http://apps.who.int/iris/bitstream/10665/44785/1/9789241502924_eng.pdf. Accessed 15 Dec 2014.

[74] Craig P, Hegglin D, Lightowlers M, Torgerson P, Wang Q (2017). Chapter 2 Echinococcosis. Control and prevention Adv Parasitol.

[75] Danson FM, Craig PS, Man W, Shi D, Giraudoux P (2004). Landscape dynamics and risk modeling of human alveolar echinococcosis. Photogramm Eng Remote Sensing.

[76] Li J, Zheng G, Liu H, Wang L, Tang Z, Shi H, et al. Situation analysis of Ningxia Province. In: China Climate Change Partnership Framework - Enhanced strategies for climate-proofed and environmentally sound agricultural production in the Yellow River Basin (C-PESAP) 2008. http://www.fao.org/fileadmin/templates/cpesap/Data/Ningxia/SASNingxiawp.pdf. Accessed 22 Mar 2016.

[77] Li Y, Conway D, Wu Y, Gao Q, Rothausen S, Xiong W (2013). Rural livelihoods and climate variability in Ningxia, Northwest China. Clim Chang.

[78] Rausch R. Life cycle patterns and geographic distribution of *Echinococcus* species. *Echinococcus* and hydatid disease. Wallingford: CAB International; 1995.

[79] Kelt DA (2000). Small mammal communities in rainforest fragments in central southern Chile. Biol Conserv.

[80] Cox MP, Dickman CR, Hunter J. Effects of rainforest fragmentation on non-flying mammals of the eastern Dorrigo Plateau, Australia. Biol Conserv. 2004;115(2):175–89.

[81] Pardini R (2004). Effects of forest fragmentation on small mammals in an Atlantic Forest landscape. Biodivers Conserv.

[82] Craig P, Giraudoux P, Shi D, Bartholomot B, Barnish G, Delattre P (2000). An epidemiological and ecological study of human alveolar echinococcosis transmission in south Gansu, China. Acta Trop.

[83] Viel J-F, Giraudoux P, Abrial V, Bresson-Hadni S. Water vole (*Arvicola terrestris* Scherman) density as risk factor for human alveolar echinococcosis. Am J Trop Med Hyg. 1999;61(4):559–65. 10.4269/ajtmh.1999.61.559 10548289

[84] Raoul F, Deplazes P, Nonaka N, Piarroux R, Vuitton DA, Giraudoux P (2001). Assessment of the epidemiological status of *Echinococcus multilocularis* in foxes in France using ELISA coprotests on fox faeces collected in the field. Int J Parasitol.

[85] Pleydell DR, Chrétien S (2010). Mixtures of GAMs for habitat suitability analysis with overdispersed presence/absence data. Comput Stat Data Anal.

[86] Marston CG, Danson FM, Armitage RP, Giraudoux P, Pleydell DR, Wang Q (2014). A random forest approach for predicting the presence of *Echinococcus multilocularis* intermediate host *Ochotona* spp. presence in relation to landscape characteristics in western China. Appl Geogr.

[87] Federer K, Armua-Fernandez MT, Hoby S, Wenker C, Deplazes P (2015). In vivo viability of *Echinococcus multilocularis* eggs in a rodent model after different thermo-treatments. Exp Parasitol.

[88] Moritz C, Patton JL, Conroy CJ, Parra JL, White GC, Beissinger SR (2008). Impact of a century of climate change on small-mammal communities in Yosemite National Park, USA. Science.

[89] Myers P, Lundrigan BL, Hoffman SM, Haraminac AP, Seto SH (2009). Climate-induced changes in the small mammal communities of the northern Great Lakes region. Glob Chang Biol.

[90] Hersteinsson P, Macdonald DW. Interspecific competition and the geographical distribution of red and arctic foxes *Vulpes vulpes* and *Alopex lagopus*. Oikos. 1992:505–15.

[91] Jenkins EJ, Schurer JM, Gesy KM (2011). Old problems on a new playing field: helminth zoonoses transmitted among dogs, wildlife, and people in a changing northern climate. Vet Parasitol.

[92] Hamm NA, Magalhaes RJS, Clements AC (2015). Earth observation, spatial data quality, and neglected tropical diseases. PLoS Negl Trop Dis.

[93] Eckert J, Deplazes P (1999). Alveolar echinococcosis in humans: the current situation in central Europe and the need for countermeasures. Parasitol Today.

[94] Hong Y, Li X, Stanton B, Lin D, Fang X, Rong M, Wang J (2006). Too costly to be ill: health care access and health seeking behaviors among rural-to-urban migrants in China. World Health Popul.

[95] Xu B, Fochsen G, Xiu Y, Thorson A, Kemp J, Jiang Q. Perceptions and experiences of health care seeking and access to TB care - a qualitative study in rural Jiangsu Province, China. Health Policy. 2004;69(2):139–49. 10.1016/j.healthpol.2003.11.006 15212861

[96] Deplazes P, Rinaldi L, Rojas CA, Torgerson P, Harandi M, Romig T, et al. Global Distribution of Alveolar and Cystic Echinococcosis. Adv Parasitol. 2017;95:315-493. 10.1016/bs.apar.2016.11.001 28131365

[97] Vuitton DA (2003). The ambiguous role of immunity in echinococcosis: protection of the host or of the parasite?. Acta Trop.

[98] Stehman SV (2009). Sampling designs for accuracy assessment of land cover. Int J Remote Sens.

[99] Hofstra N, Haylock M, New M, Jones P, Frei C. Comparison of six methods for the interpolation of daily, European climate data. J Geophys Res Atmos. 2008;113(D21)

[100] Ashraf M, Loftis JC, Hubbard K (1997). Application of geostatistics to evaluate partial weather station networks. Agric For Meteorol.

