# Supplementary figures and images for: Spatiotemporal patterns and environmental drivers of human echinococcoses over a twenty-year period in Ningxia Hui Autonomous Region, China
Source: Parasit Vectors. 2018 Feb 22;11:108. doi: 10.1186/s13071-018-2693-z (PMC5824458; doi:10.1186/s13071-018-2693-z)

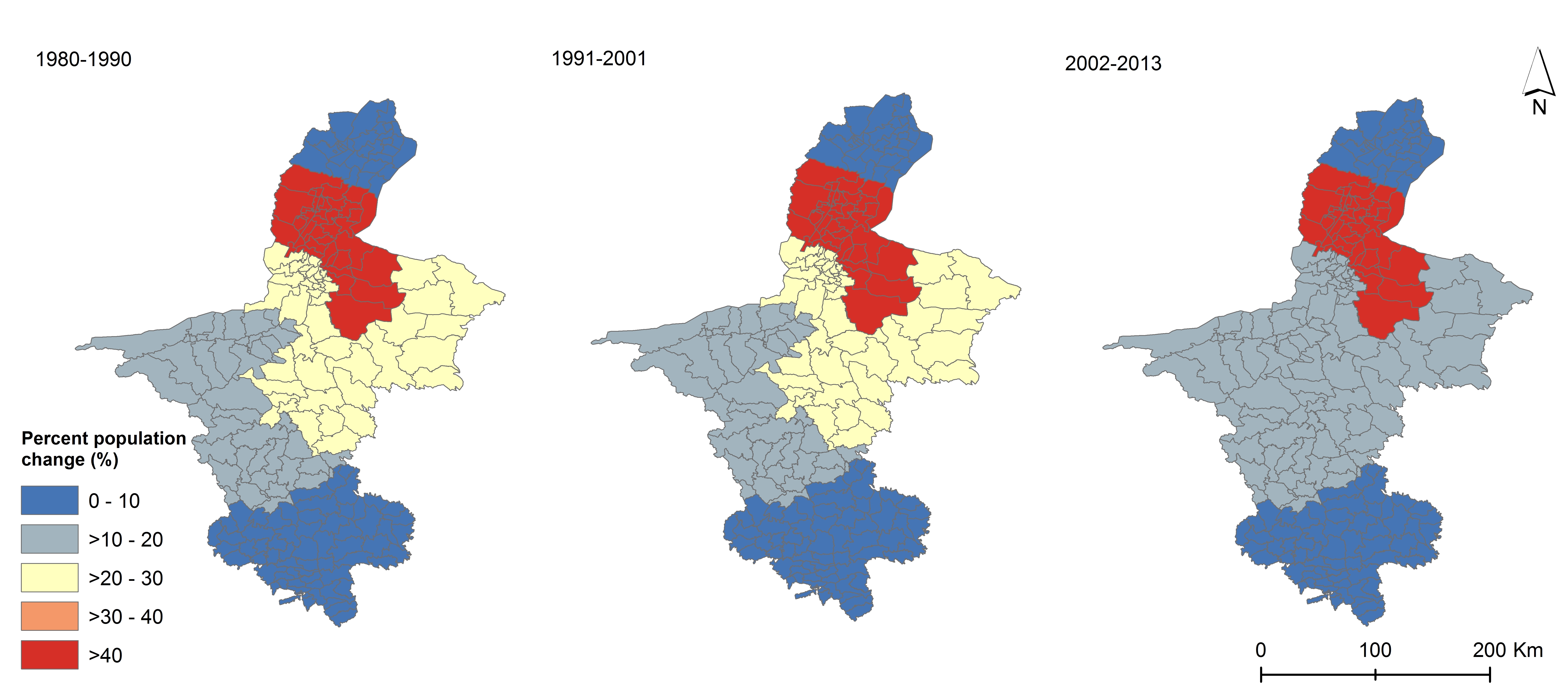

Supplement: Supplementary file 1 — Percent population change in NHAR for the periods 1980–1990, 1991–2001 and 2002–2013. (JPEG 3042 kb) [file 13071_2018_2693_MOESM1_ESM.jpg]

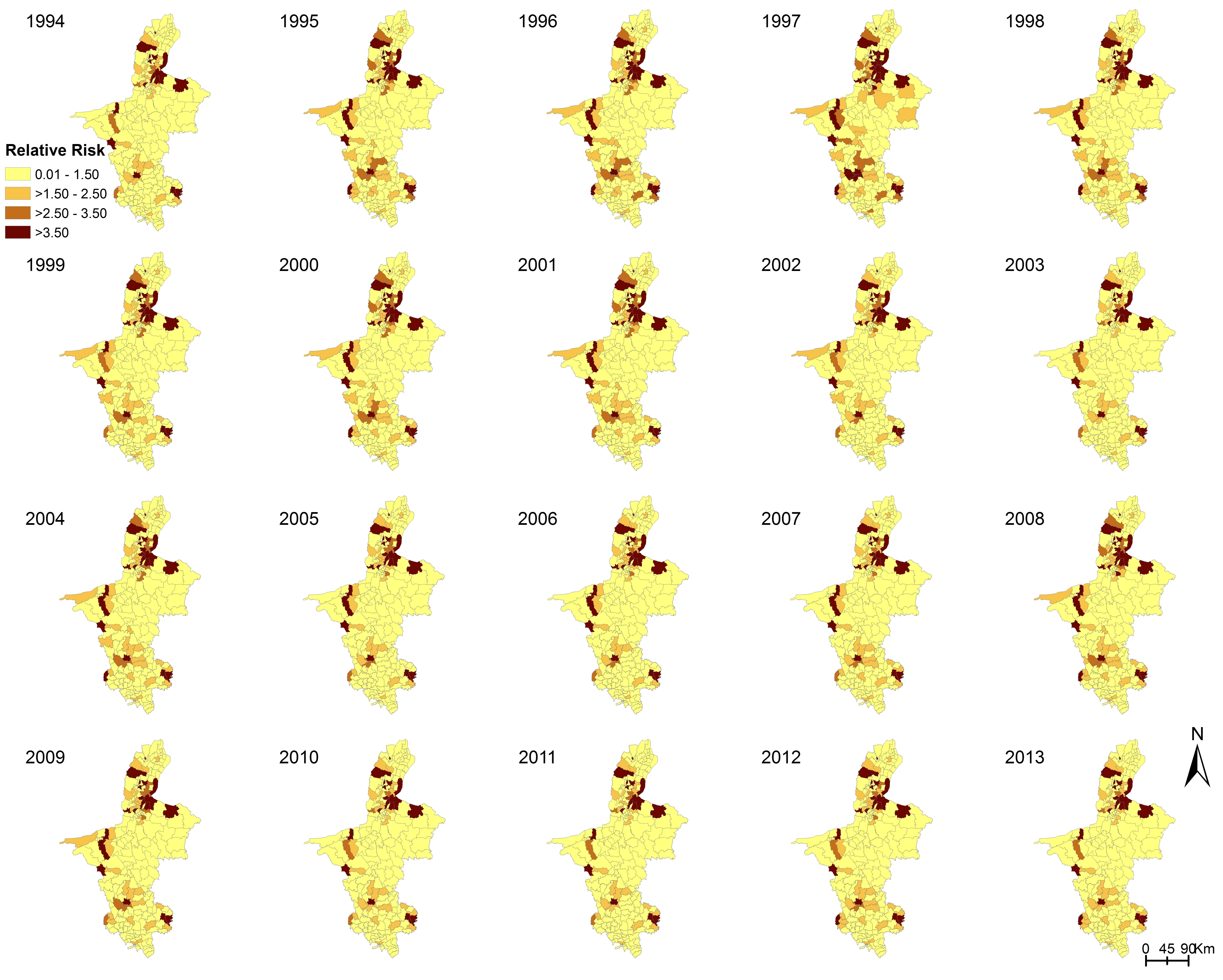

Supplement: Supplementary file 16 — Spatial distribution of annual raw relative risks for CE in NHAR for the period 1994 to 2013. (JPEG 7859 kb) [file 13071_2018_2693_MOESM16_ESM.jpg]

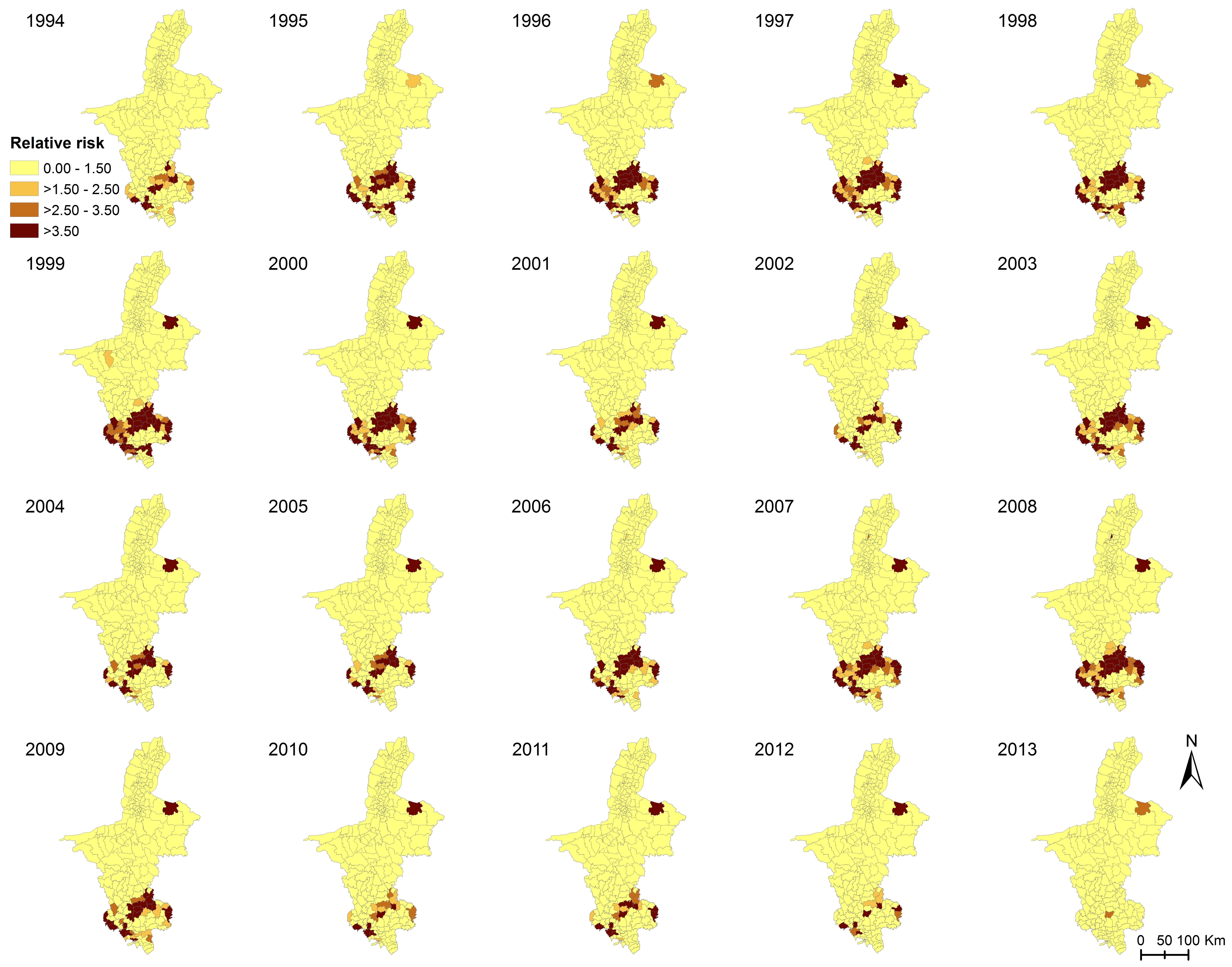

Supplement: Supplementary file 17 — Spatial distribution of annual relative risks for AE in NHAR for the period 1994 to 2013. (JPEG 7722 kb) [file 13071_2018_2693_MOESM17_ESM.jpg]
