# Supplementary material for: Spatiotemporal patterns and environmental drivers of human echinococcoses over a twenty-year period in Ningxia Hui Autonomous Region, China
Source: Parasit Vectors. 2018 Feb 22;11:108. doi: 10.1186/s13071-018-2693-z (PMC5824458; doi:10.1186/s13071-018-2693-z)
Supplement: Supplementary file 2 — Spatial distribution of the average annual mean temperature in °C in NHAR for the period 1980–2013. (DOCX 209 kb) [file 13071_2018_2693_MOESM2_ESM.docx]

**Additional file 2:** Spatial distribution of the average annual mean temperature in °C in NHAR for the period 1980–2013.

**
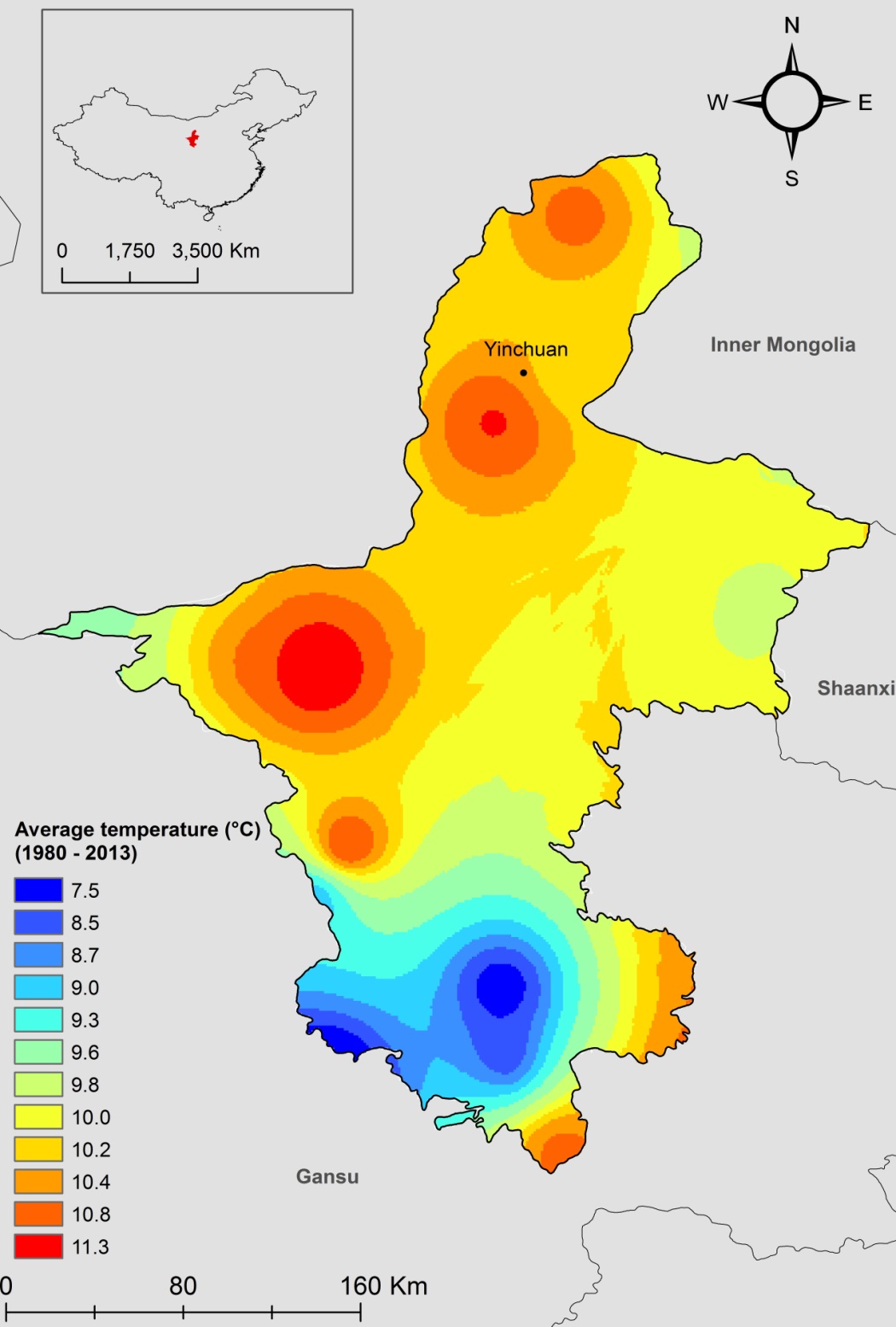
**
