# Supplementary material for: Spatiotemporal patterns and environmental drivers of human echinococcoses over a twenty-year period in Ningxia Hui Autonomous Region, China
Source: Parasit Vectors. 2018 Feb 22;11:108. doi: 10.1186/s13071-018-2693-z (PMC5824458; doi:10.1186/s13071-018-2693-z)

**Additional file 4:** Maps of the spatial distribution of **a** annual, **b** summer and **c** winter temperature trends, and **d** annual, **e** summer and **f** winter precipitation trends in NHAR for the period 1 January 1980 to 31 December 2013. Note, the values presented in the figure are relative to the provincial average per decade.


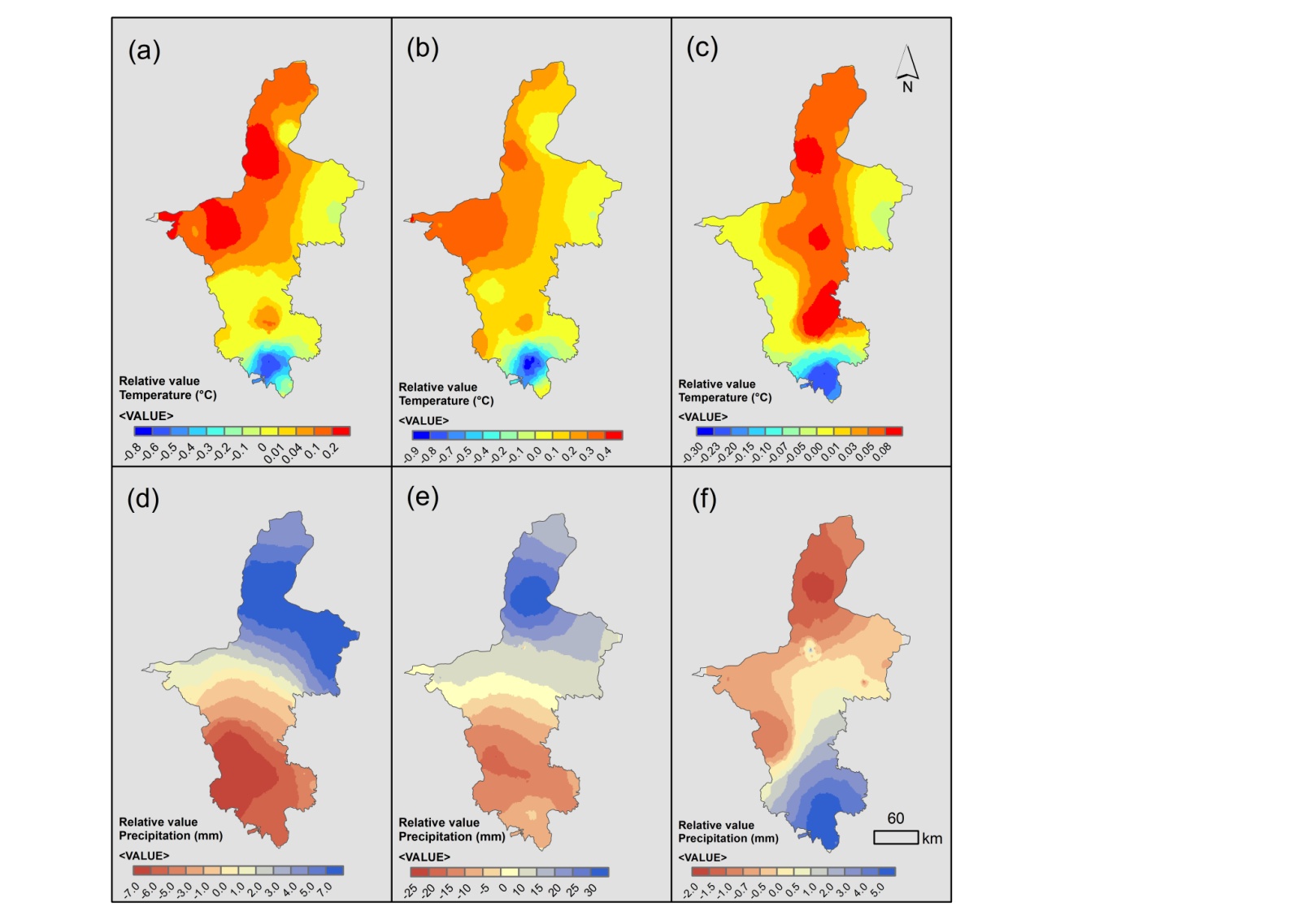

Supplement: Supplementary file 4 — Maps of the spatial distribution of a annual, b summer and c winter temperature trends, and d annual, e summer and f winter precipitation trends in NHAR for the period 1 January 1980 to 31 December 2013. Note, the values presented in the figure are relative to the provincial average per decade. (DOCX 244 kb) [file 13071_2018_2693_MOESM4_ESM.docx]
