# Supplementary material for: Spatiotemporal patterns and environmental drivers of human echinococcoses over a twenty-year period in Ningxia Hui Autonomous Region, China
Source: Parasit Vectors. 2018 Feb 22;11:108. doi: 10.1186/s13071-018-2693-z (PMC5824458; doi:10.1186/s13071-018-2693-z)
Supplement: Supplementary file 5 — OpenBUGS code used to develop the Bayesian spatial model (Model II) for cystic echinococcosis in NHAR from 1 January 1994 to 31 December 2013. (DOCX 13 kb) [file 13071_2018_2693_MOESM5_ESM.docx]

**Additional file 5:** OpenBUGS code used to develop the Bayesian spatial model (Model II) for cystic echinococcosis in NHAR from 1 January 1994 to 31 December 2013.

model{

s[1:227] ~ car.normal(adj[], weights[], num[], tau.s);

for (k in 1:sumNumNeigh){

weights[k] <- 1

}

for(i in 1:4540){

Cases[i]~dpois(mu[i])

log(mu[i])<-log.RR[i] + log(Exp[i])

log.RR[i]<-alpha +s[Township[i]] + beta[1]*Tmean[i] + beta[2]*T_winmean[i] + beta[3]*herb[i] + beta[4]*MA5bare[i] + beta[5]*MA5Cult[i] +beta[6]*MA5Forest[i] + beta[7]*MA5Shrub[i] + beta[8]*MA6Water[i] + beta[9]*MA2Tmean[i] + beta[10]*MA4Pmin[i] +beta[11]* pow(Tmean[i],2) + beta[12]*Time[i]

RR[i]<-exp(log.RR[i])

}

for(i in 1:227){

#u[i]~dnorm(0,tau.u)

}

alpha~dflat()

for(i in 1:12){

beta[i]~dnorm(0,0.001)

}

tau.s~dgamma(0.5,0.0005)

}

list(alpha=0,beta=c(0,0,0,0,0,0,0,0,0,-0.1,-0.1,-0.1), tau.s=0.5)
