# Supplementary material for: Spatiotemporal patterns and environmental drivers of human echinococcoses over a twenty-year period in Ningxia Hui Autonomous Region, China
Source: Parasit Vectors. 2018 Feb 22;11:108. doi: 10.1186/s13071-018-2693-z (PMC5824458; doi:10.1186/s13071-018-2693-z)

**Additional file 8:** Number of observed and expected number of alveolar echinococcosis (AE) cases by year (1994–2013) in NHAR for the period 1 January 1994 to 31 December 2013.


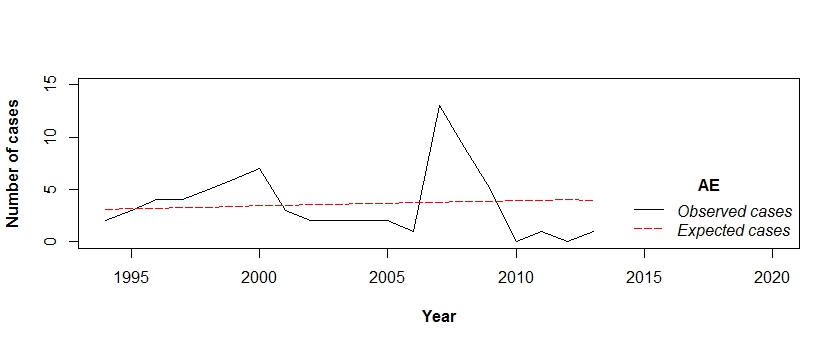

Supplement: Supplementary file 8 — Number of observed and expected number of AE cases by year (1994–2013) in NHAR for the period 1 January 1994 to 31 December 2013. (DOCX 72 kb) [file 13071_2018_2693_MOESM8_ESM.docx]
