# Supplementary material for: Spatiotemporal patterns and environmental drivers of human echinococcoses over a twenty-year period in Ningxia Hui Autonomous Region, China
Source: Parasit Vectors. 2018 Feb 22;11:108. doi: 10.1186/s13071-018-2693-z (PMC5824458; doi:10.1186/s13071-018-2693-z)
Supplement: Supplementary file 9 — Annual temperature in NHAR for the period 1 January 1980 to 31 December 2013 and number of cases of CE and AE for the period 1 January 1994 to 31 December 2013. (DOCX 98 kb) [file 13071_2018_2693_MOESM9_ESM.docx]

**Additional file 9:** Annual temperature in NHAR for the period 1 January 1980 to 31 December 2013 and number of cases of CE and AE for the period 1 January 1994 to 31 December 2013.

**
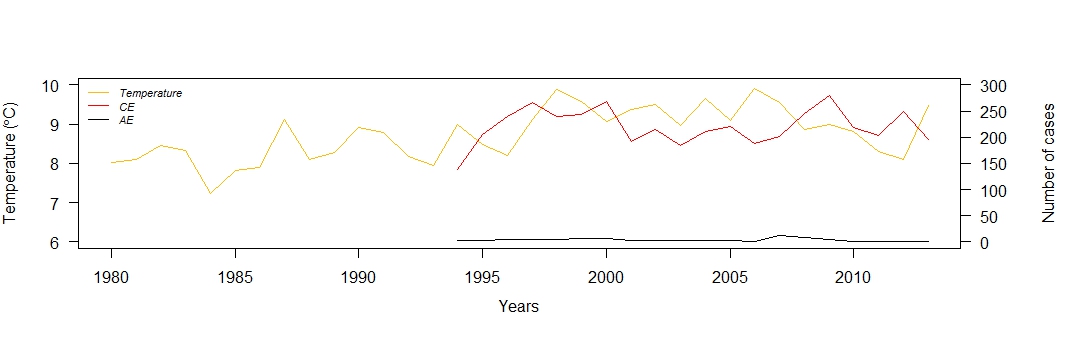
**
