# Supplementary material for: Spatiotemporal patterns and environmental drivers of human echinococcoses over a twenty-year period in Ningxia Hui Autonomous Region, China
Source: Parasit Vectors. 2018 Feb 22;11:108. doi: 10.1186/s13071-018-2693-z (PMC5824458; doi:10.1186/s13071-018-2693-z)

**Additional file 15:** Scatterplots of number of AE cases by township against annual mean temperature calculated for the period 11–15 years before diagnosis.


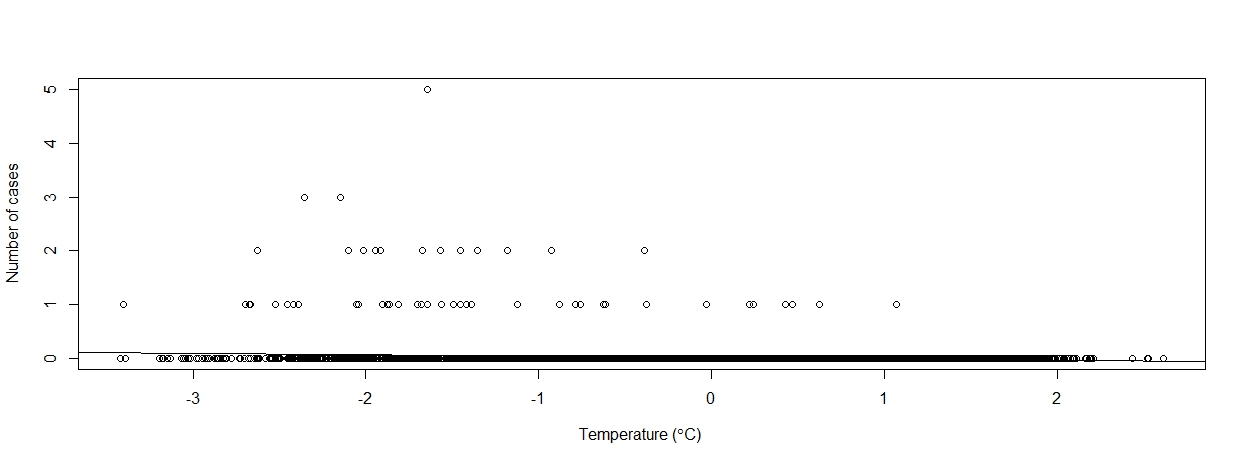

Supplement: Supplementary file 15 — Scatterplots of number of AE cases by township against annual mean temperature calculated for the period 11–15 years before diagnosis. (DOCX 72 kb) [file 13071_2018_2693_MOESM15_ESM.docx]
